# Towards collaborative data science in mental health research: The ECNP neuroimaging network accessible data repository

**DOI:** 10.1016/j.nsa.2024.105407

**Published:** 2024-12-09

**Authors:** Adyasha Khuntia, Madalina-Octavia Buciuman, John Fanning, Aleks Stolicyn, Clara Vetter, Reetta-Liina Armio, Tiina From, Federica Goffi, Lisa Hahn, Tobias Kaufmann, Heikki Laurikainen, Eleonora Maggioni, Ignacio Martinez-Zalacain, Anne Ruef, Mark Sen Dong, Emanuel Schwarz, Letizia Squarcina, Ole Andreassen, Marcella Bellani, Paolo Brambilla, Neeltje van Haren, Jarmo Hietala, Stephen M. Lawrie, Carles Soriano-Mas, Heather Whalley, Maxime Taquet, Eva Meisenzahl, Peter Falkai, Ariane Wiegand, Nikolaos Koutsouleris, for the ECNP Neuroimaging Network

**Affiliations:** a Department of Psychiatry and Psychotherapy, University Hospital, Ludwig-Maximilian University, Munich, Germany; b International Max-Planck School for Translational Psychiatry, Germany Max-Planck Institute of Psychiatry Munich, Munich, Germany; c Max Planck Institute of Psychiatry, Munich, Germany; d German Center for Mental Health, Partner Site Munich, Augsburg, Germany; e Division of Psychiatry, Centre for Clinical Brain Sciences, University of Edinburgh, Edinburgh, Scotland, UK; f Munich Center of Machine Learning, Munich, Germany; g Helmholtz Association - Munich School for Data Science, Munich, Germany; h Department of Psychiatry, University of Turku, Turku, Finland; i Turku PET Centre, University of Turku, Turku, Finland; j Turku University Hospital, Turku, Finland; k Department of Electronics, Information and Bioengineering, Politecnico di Milano, Milan, Italy; l Department of Neurosciences and Mental Health, Fondazione IRCCS Ca' Granda Ospedale Maggiore Policlinico, University of Milan, Milan, Italy; m NORMENT Centre, Division of Mental Health and Addiction, Oslo University Hospital, Oslo, Norway; n Department of Psychiatry and Psychotherapy, University of Tübingen, Germany; o German Center for Mental Health, Partner Site Tübingen, Germany; p Bellvitge Biomedical Research Institute-IDIBELL, Psychiatry Department, Bellvitge University Hospital, CIBERSAM and Department of Psychobiology and Methodology of Health Sciences, Universitat Autònoma de Barcelona, Spain; q Department of Radiology, Bellvitge University Hospital, Barcelona, Spain; r Hector Institute for Artificial Intelligence in Psychiatry, Central Institute of Mental Health, Medical Faculty Mannheim, Heidelberg University, Mannheim, Germany; s Department of Psychiatry and Psychotherapy, Central Institute of Mental Health, Medical Faculty Mannheim, Heidelberg University, Mannheim, Germany; t German Center for Mental Health, Partner Site Mannheim-Heidelberg-Ulm, Germany; u Department of Pathophysiology and Transplantation, University of Milan, Milan, Italy; v Section of Psychiatry, Dept of Neurosciences, Biomedicine and Movement, University of Verona, Verona, Italy; w Department of Neurosciences and Mental Health, Fondazione IRCCS Ca' Granda Ospedale Maggiore Policlinico, Milan, Italy; x Department of Child and Adolescent Psychiatry/Psychology, Erasmus Medical Centre, Rotterdam, the Netherlands; y Department of Psychiatry, University Medical Centre Utrecht, Utrecht, the Netherlands; z CIBERSAM, Carlos III Health Institute, Madrid, Spain; aa Bellvitge Biomedical Research Institute-IDIBELL, Department of Psychiatry, Bellvitge University Hospital, Barcelona, Spain; ab Department of Social Psychology and Quantitative Psychology, Institute of Neurosciences, University of Barcelona, Spain; ac Department of Psychiatry, University of Oxford, Oxford, UK; ad Oxford Health NHS Foundation Trust, Oxford, UK; ae Department of Psychiatry and Psychotherapy, Medical Faculty, Heinrich-Heine University, Düsseldorf, Germany; af Institute of Psychiatry, Psychology & Neuroscience, King’s College London, London, UK

**Keywords:** Multi-site data, Transdiagnostic, Machine learning, Data repository, Psychiatry, Neuroimaging, Structural MRI

## Abstract

The current biologically uninformed psychiatric taxonomy complicates optimal diagnosis and treatment. Neuroimaging-based machine learning methods hold promise for tackling these issues, but large-scale, representative cohorts are required for building robust and generalizable models. The European College of Neuropsychopharmacology Neuroimaging Network Accessible Data Repository (ECNP-NNADR) addresses this need by collating multi-site, multi-modal, multi-diagnosis datasets that enable collaborative research. The newly established ECNP-NNADR includes 4829 participants across 21 cohorts and 11 distinct psychiatric diagnoses, available via the Virtual Pooling and Analysis of Research data (ViPAR) software. The repository includes demographic and clinical information, including diagnosis and questionnaires evaluating psychiatric symptomatology, as well as multi-atlas grey matter volume regions of interest (ROI). To illustrate the opportunities offered by the repository, two proof-of-concept analyses were performed: (1) multivariate classification of 498 patients with schizophrenia (SZ) and 498 matched healthy control (HC) individuals, and (2) normative age prediction using 1170 HC individuals with subsequent application of this model to study abnormal brain maturational processes in patients with SZ. In the SZ classification task, we observed varying balanced accuracies, reaching a maximum of 71.13% across sites and atlases. The normative-age model demonstrated a mean absolute error (MAE) of 6.95 years [coefficient of determination (*R*^*2*^) = 0.77, *P* < .001] across sites and atlases. The model demonstrated robust generalization on a separate HC left-out sample achieving a MAE of 7.16 years [*R*^*2*^ = 0.74,*P* < .001]. When applied to the SZ group, the model exhibited a MAE of 7.79 years [*R*^*2*^ = 0.79, *P* < .001], with patients displaying accelerated brain-aging with a brain age gap (BrainAGE) of 4.49 (8.90) years. Conclusively, this novel multi-site, multi-modal, transdiagnostic data repository offers unique opportunities for systematically tackling existing challenges around the generalizability and validity of imaging-based machine learning applications for psychiatry.

## 1 Introduction

The global prevalence of mental disorders has dramatically increased over the past two decades (GBD, 2019 Mental Disorders Collaborators, 2022). Recent reports indicate that, as of 2020, approximately one billion individuals worldwide suffer from mental disorders, impacting the global economy by approximately 2.5 trillion dollars annually; this economic burden is forecasted to reach 6 trillion dollars by 2030 (The Lancet Global Health, 2020; Chodavadia et al., 2023). Considerable obstacles exist for the accurate, early diagnosis of these complex disorders, owing to the heterogeneity of the established “biology-uninformed” taxonomic system which does not consider pathophysiological signals and thus hinder the identification of pathognomonic biopsychosocial patterns (Fusar-Poli et al., 2016; McClintock et al., 2010).

Moreover, classical inferential statistics, which have been commonly used in psychiatric research to date (Bzdok, 2017), have significant shortcomings. These methods are limited in their ability to extract predictive patterns from multimodal, heterogeneous data sets, and to embed these patterns into scalable tools for individualized patient management in real-world clinical care (Dwyer et al., 2018; Dwyer and Koutsouleris, 2022).

To address these shortcomings, the field of precision psychiatry has increasingly embraced machine learning (ML) techniques as a promising methodological avenue for dealing with multi-scale, high-dimensional, and heterogeneous datasets. Specifically, supervised ML methods offer the possibility to improve early recognition, differential diagnosis, and inform treatment selection based on classification and regression algorithms, whereas unsupervised ML techniques facilitate a biologically informed revision of existing diagnostic concepts via clustering and factorization methods (Dwyer et al., 2018; Bzdok and Meyer-Lindenberg, 2018; Zhou et al., 2022). However, despite the promise of this methodological revolution in psychiatric research, there are caveats that need to be addressed prior to clinical translation. Concretely, model generalizability beyond the initial discovery sample is a key translational requirement that must be unequivocally demonstrated. However, meeting this criterion requires large and heterogenous samples that are representative of the inherent heterogeneity of the mental disorder in question (Lombardo et al., 2019; Feczko et al., 2019). Furthermore, model specificity and applicability must be measured from a transdiagnostic perspective to establish the “operational windows” of population characteristics, such as age, sex, and ethnicity, that conjointly determine model performance and thus model eligibility for specific patient populations. Collaborative, multi-site, multi-project data repositories provide an opportunity to address these intertwined issues and facilitate the translation of psychiatric research into clinically informative tools.

To address this important need, we introduce the European College of Neuropsychopharmacology Neuroimaging Network Accessible Data Repository (ECNP-NNADR) project as a new collaborative data science initiative. The project has so far produced an accessible data repository containing clinical and magnetic resonance imaging (MRI) data safely shared using the Virtual Pooling and Analysis of Research data (ViPAR) software (Kw et al., 2015). The initiative started in October 2020 with seven European sites, all of which have contributed patient data comprising different psychiatric disorders (N = 2363), including schizophrenia (SZ), major depressive disorder (MDD), bipolar disorder I (BDI), bipolar disorder II (BDII), borderline personality disorder (BPD), obsessive compulsive disorder (OCD), mild cognitive impairment (MCI), hoarding disorder (HD), general anxiety disorder (GAD), social phobia (SP), clinical high-risk for psychosis (CHR) and first-episode psychosis (FEP), and large samples of healthy control (HC) individuals (N = 2466). The goal of the ECNP-NNADR initiative is to create a multi-site, multi-variable data repository that facilitates collaborative psychiatric research, and offer the opportunity to identify and validate complex disease patterns through the simultaneous investigation of different data modalities.

In the following section, we describe the data collection, harmonization, and sharing process across different sites, as well as the challenges encountered. Moreover, we describe two proof-of-concept analyses which were conducted to demonstrate the feasibility of using state-of-the-art ML methods in such a collaborative setup. By applying support vector machines (SVM) to volumetric region-of-interest (ROI) measures derived from structural MRI data, we first developed a classifier that separates SZ patients from HC. Secondly, we used a normative approach to train a regression model to predict age based on ROI data from HC, which we then applied to SZ patients to evaluate clinical effects on brain aging. Furthermore, we evaluated how brain age gap (BrainAGE) scores are related to HC and SZ separability in the classification analyses. These analysis tasks were selected based on the ample literature available for comparison and with the aim to demonstrate the potential of the current data repository and its adjunct analytical facilities for future multi-modal, multi-site machine learning studies.

## 2 Methods

### 2.1 Description of the ECNP-NNADR cohorts

To date, seven European sites representing six different countries participate in the ECNP-NNADR project: The Ludwig-Maximilian University Hospital in Munich, Bavaria, Germany (MUC); NORMENT Centre, Division of Mental Health and Addiction, Oslo University, Oslo, Norway (OSL); Bellvitge Biomedical Research Institute-IDIBELL, Universitat de Barcelona, Barcelona, Spain (BAR); University of Edinburgh, Edinburgh, Scotland; Università degli Studi di Milano, Milan, Italy (MIL); University of Turku, Turku, Finland (TUR); Università degli Studi di Verona, Verona, Italy (VER). Across the participating sites, patients were included if they had complete demographic information including age, sex, diagnosis, and at least one complete score set in one of the clinical scales or MRI data. For more details regarding the inclusion/exclusion criteria used for each of the cohorts included in the ECNP-NNADR, see Table S1. All included studies were approved by their respective local ethics committees (Table S1).

**Fig. 1:**
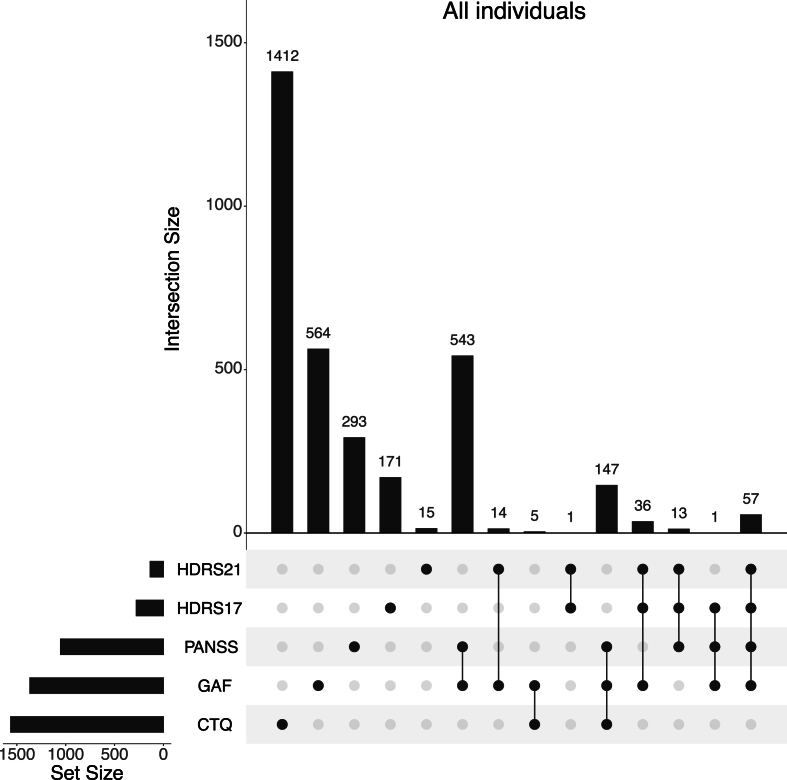
**Overlap of clinical scales available for each participant within the different diagnostic groups.** Connecting lines denote overlap in the questionnaires. The bars represent the total sample size for each of the questionnaires. Set size refers to the overall total sample size for the respective questionnaires, and interaction size refers to the number of samples for that combination of questionnaires. CTQ = Childhood Trauma Questionnaire, GAF = Global Assessment of Functioning, HDRS17 = Hamilton Depression Rating Scale (17 items), HDRS21 = Hamilton Depression Rating Scale (21 items), MADRS = Montgomery–Åsberg Depression Rating Scale, PANSS = Positive and Negative Syndrome Scale. The visualization was generated using the UpSetR package in R.

Currently, the repository consists of clinical and MRI data from 21 cohorts across the seven sites, resulting in a total sample size of 4829 participants, including HC individuals and eleven distinct psychiatric conditions. We provide an overview of the available clinical and MRI data across sites in Table 1 and across diagnoses in Table 2 and Fig. 1, and section *3.1. Repository sample characteristics*.

**Table 1:** Overview of demographic, clinical and MRI data available for each site of the ECNP-NNADR.

	Barcelona (N = 781)	Edinburgh (N = 1186)	Milan (N = 174)	Munich (N = 922)	Oslo (N = 822)	Turku (N = 334)	Verona (N = 610)
Age (yrs), N (mean ± SD)	781 (37.75 ± 15.52)	1186 (60.04 ± 10.05)	174 (40.76 ± 14.33)	922 (33.18 ± 11.45)	822 (33.61 ± 10.14)	334 (26.74 ± 6.33)	610 (38.9 ± 12.42)
Sex (male, female)	373, 408	490, 696	88, 86	477, 445	438, 384	172, 162	316, 294
Diagnoses	433 HC, 53 MDD, 218 OCD, 21 MCI, 20 HD, 32 GAD, 4 SP	820 HC, 344 MDD, 12 BDI, 10 BDII	26 HC, 110 BDI, 38 BDII	502 HC, 156 SZ, 104 MDD, 43 BDI, 59 BPD, 58 CHR	295 HC, 224 SZ, 191 BDI, 112 Other	95 HC,	295 HC, 123 SZ, 38 MDD, 74 BDI, 12 BDII, 68 FEP
						10 SZ,	
						123 MDD, 24 BDI,	
						1 BDII,	
						4 OCD,	
						8 GAD,	
						18 HD,	
						51 MCI	
HDRS17, n (mean ± SD)	92 (6.76 ± 7.37)	–	–	79 (21.66 ± 5.95)	–	–	108 (9.26 ± 6.95)
HDRS21, n (mean ± SD)	–	–	29 (7.66 ± 8.23)	–	–	–	107 (10.83 ± 8.61)
MADRS, n (mean ± SD)	3 (35.67 ± 8.08)	–	13 (3.00 ± 3.49)	41 (17.41 ± 9.06)	–	–	–
CTQ, n (mean ± SD)	75 (39.95 ± 13.13)	1157 (34.01 ± 11.70)	–	180 (31.32 ± 6.47)	–	152 (37.35 ± 10.48)	–
GAF, n (mean ± SD)	–	–	95 (33.73 ± 15.23)	65 (51.17 ± 13.74)	414 (49.76 ± 11.58)	334 (62.96 ± 20.87)	459 (69.74 ± 16.65)
PANSS, n (mean ± SD)	–	–	–	239 (76.21 ± 27.57)	406 (53.22 ± 16.22)	180 (45.56 ± 19.23)	229 (64.89 ± 16.21)
MRI atlas data, N	781	1186	175	922	822	241	610

*Note*. yrs = years, SD = standard deviation, SZ = schizophrenia, MDD = major depressive disorder, BDI = bipolar disorder I, BDII = bipolar disorder II, BPD = borderline personality disorder, OCD = obsessive compulsive disorder, MCI = mild cognitive impairment, HD = hoarding disorder, GAD = general anxiety disorder, SP = social phobia, CHR = clinical high-risk of schizophrenia, HDRS17 = Hamilton Depression Rating Scale (17 items), HDRS21 = Hamilton Depression Rating Scale (21 items), MADRS = Montgomery-Åsberg Depression Rating Scale, CTQ = Childhood Trauma Questionnaire, GAF = Global Assessment of Functioning, PANSS = Positive and Negative Syndrome Scale.

**Table 2:** Overview of demographic, clinical and MRI variables grouped by diagnosis.

	HC (N = 2466)	SZ (N = 513)	MDD (N = 662)	BDI (N = 454)	BDII (N = 61)	BPD (N = 59)	OCD (N = 222)	MCI (N = 72)	HD (N = 38)	GAD (N = 40)	SP (N = 4)	CHR (N = 58)	FEP (N = 68)	Other (N = 112)
Age (yrs), N (mean ± SD)	2466 (42.93 ± 16.91)	513 (33.46 ± 10.69)	662 (49.76 ± 15.88)	454 (39.19 ± 13.38)	61 (48.23 ± 4.16)	59 (25.93 ± 6.98)	222 (36.09 ± 10.27)	72 (39.61 ± 21.19)	38 (38.53 ± 14.39)	40 (25.62 ± 3.99)	4 (23.50 ± 3.00)	58 (24.48 ± 5.56)	68 (31.10 ± 9.24)	112 (30.75 ± 9.99)
Sex (male, female)	1235, 1231	299, 214	256, 406	209,246	31, 30	0, 59	106, 116	40, 32	25, 13	17, 23	3, 1	36, 22	31, 37	66, 46
HDRS17, N (mean ± SD)	31 (1.29 ± 2.80)	9 (8.89 ± 7.51)	149 (17.36 ± 7.88)	50 (7.60 ± 6.82)	12 (9.08 ± 5.60)	–	–	21 (3.76 ± 2.93)	–	–	–	–	7 (8.29 ± 7.89)	–
HDRS21, N (mean ± SD)	–	8 (7.51 ± 4.62)	30 13.90 ± 7.49)	69 (9.09 ± 8.07)	22 (7.00 ± 5.67)	–	–	–	–	–	–	–	7 (9.43 ± 9.20)	–
MADRS, N (mean ± SD)	–	–	3 (35.67 ± 8.08)	4 (2.75 ± 4.86)	9 (3.11 ± 3.06)	–	–	–	–	–	–	41 (17.41 ± 9.06)	–	–
CTQ, N (mean ± SD)	1039 (31.5 ± 7.92)	5 (38.80 ± 10.47)	391 (39.47 ± 14.72)	20 (45.40 ± 16.76)	11 (48.00 ± 19.60)	–	76 (39.89 ± 13.05)	14 (37.64 ± 9.40)	5 (41.00 ± 5.78)	3 (39.00 ± 13.00)	–	–	–	–
GAF, N (mean ± SD)	66 (78.54 ± 5.76)	283 (47.76 ± 12.41)	156 (55.44 ± 11.46)	312 (50.01 ± 16.38)	26 (42.19 ± 18.00)	–	4 (48.00 ± 8.12)	51 (47.22 ± 15.14)	18 (54.39 ± 13.64)	8 (60.75 ± 9.65)	–	41 (59.15 ± 10.36)	–	68 (50.01 ± 11.41)
PANSS, N (mean ± SD)	–	433 (71.72 ± 27.07)	92 (52.61 ± 16.49)	200 (47.3 ± 11.40)	13 (60.15 ± 3.27)	43 (70.65 ± 18.60)	1 (68 ± 0)	17 (62.71 ± 18.84)	6 (60.50 ± 29.66)	3 (39.33 ± 4.51)	–	44 (60.27 ± 18.60)	59 (56.78 ± 13.97)	77 (53.38 ± 14.18)
MRI atlas data, N	2466	513	662	455	61	59	222	72	38	40	4	58	68	112

*Note*. yrs = years, SD = standard deviation, SZ = schizophrenia, MDD = major depressive disorder, BDI = bipolar disorder I, BDII = bipolar disorder II, BPD = borderline personality disorder, OCD = obsessive compulsive disorder, MCI = mild cognitive impairment, HD = hoarding disorder, GAD = general anxiety disorder, SP = social phobia, CHR = clinical high-risk of schizophrenia, HDRS17 = Hamilton Depression Rating Scale (17 items), HDRS21 = Hamilton Depression Rating Scale (21 items), MADRS = Montgomery-Åsberg Depression Rating Scale, CTQ = Childhood Trauma Questionnaire, GAF = Global Assessment of Functioning, PANSS = Positive and Negative Syndrome Scale.

### 2.2 Data harmonization

A significant challenge in creating big data repositories consists of the appropriate harmonization of data across cohorts and study sites to facilitate the analytical process. Within the ECNP-NNADR, we harmonized the clinical and MRI data based on modality-specific data dictionaries, defining the specific data type, scale (continuous/categorical), and validity range for each variable, parameters which were followed by all participating sites. Furthermore, a quality control script was provided to all the sites for checking the value ranges of all variables according to the dictionaries prior to sharing the data within the ViPAR platform.

#### 2.2.1 Clinical data

The clinical dictionary consisted of demographic variables such as the cohort, age, sex, and site, as well as clinical parameters, including the psychiatric diagnosis and six different clinical scales common across sites (GBD, 2019 Mental Disorders Collaborators, 2022): Hamilton Depression Rating Scale 17 (HDRS 17) (The Lancet Global Health, 2020), Hamilton Depression Rating Scale 21 (HDRS 21) (Chodavadia et al., 2023), Montgomery-Åsberg Depression Rating Scale (MADRS) (Fusar-Poli et al., 2016), Childhood Trauma Questionnaire (CTQ) (McClintock et al., 2010), Global Assessment of Functioning (GAF), and (Bzdok, 2017) Positive and Negative Syndrome Scale (PANSS). For a more detailed description of the clinical scales and their coding systems in the ECNP-NNADR clinical dictionary, refer to section *1.1* of the *Supplementary Methods*.

#### 2.2.2 MRI data acquisition and preprocessing

The ECNP-NNADR represents a collaborative effort aimed at creating a comprehensive neuroimaging-based database by aggregating data from existing, separately conducted neuroimaging studies. Hence, it is important to note that the MRI data collection was not harmonized a priori in terms of MRI parameters across different sites. Detailed descriptions of the MRI parameters utilized at each site are available within the repository (Table S2).

The MRI image processing was harmonized across sites, such that the T1-weighted structural images were processed using the morphometric analysis pipeline implemented in CAT12 (version 12.8, optimized for multi-site deployment; https://neuro-jena.github.io/enigma-cat12/). MATLAB-based scripts and instruction files were developed at LMU and were designed to cater to the needs of all participating sites, offering both standalone versions that do not require a MATLAB license or standard versions. By distributing these resources to all sites, we aimed to streamline and facilitate the harmonization process of MRI image processing, thereby contributing to the reliability and comparability of our neuroimaging analyses. The pipeline produced grey matter volume (GMV) and white matter volume (WMV) measures for a set of ROIs parcellated according to the Schaefer-200 (GMV and WMV) (Koutsouleris et al., 2021), AAL3 (GMV) (Bischl et al., 2016), and Hammers (GMV and WMV) atlases (Thölke et al., 2023).

### 2.3 Data sharing using ViPAR

To comply with different data privacy regulations imposed by the data-providing institutions, the ECNP-NNADR project employs the ViPAR software (Kw et al., 2015). ViPAR has been successfully used by similar international multi-consortia initiatives employing ML methods for psychiatric applications, such as the HARMONY collaboration (Koutsouleris et al., 2021).

**Fig. 2:**
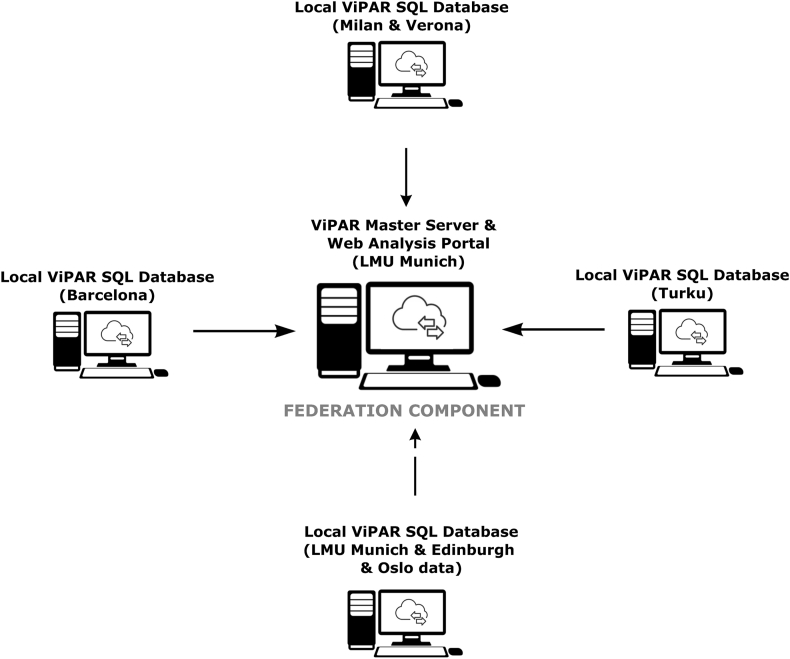
**Overview of the ViPAR-based setup used for data federation within the ECNP Neuroimaging Network.** Data of each remote site is stored locally within SQL databases synchronized with the ViPAR Master Server based on a common data dictionary. When an analysis is run through the Master Server’s Web Analysis Portal, local data from the remote sites is temporarily pooled into the Master Server’s RAM for the limited duration of the analysis, without the need for permanent central storage.

The ViPAR platform consists of an analysis portal hosted at the central master server located within a secure research-dedicated subnetwork of LMU University Hospital, and local SQL databases established at the participating sites, where the harmonized data are permanently stored (Fig. 2). Based on this setup, ViPAR allows federated, simultaneous pooling of the anonymized data from each of the local databases into the central master server’s random-access memory (RAM) for the limited run-time of a given analysis, circumventing the need to create local physical copies of the data. Currently, the ViPAR platform supports MATLAB and R software for conducting analyses, with further extensions (e.g. Python) planned based on upcoming analytical requirements. Additionally, the Edinburgh and Oslo sites shared their data directly with LMU through Data Transfer Agreements and is therefore stored within the local Munich ViPAR database.

The ViPAR web portal supports the creation of projects and assignment of researchers to projects authorized by the ECNP-NNADR steering board, such that the researchers' data access can be closely regulated, and analyses are automatically logged within the master server (Fig. 2). Currently, members of the ECNP Neuroimaging Network who made data available to the ECNP-NNADR project are eligible to submit analysis proposals, which are evaluated by the steering board of the ECNP-NNADR.

### 2.4 Proof-of-concept analyses

We performed two proof-of-concept ML-based analyses to highlight the suitability of the ECNP-NNADR for multivariate analyses. In the first analysis, we developed a multivariate classification model aimed at distinguishing between HC individuals and patients with SZ by utilizing GMV ROI data as features (see section *2.4.1. Classification analysis* for further methodological details). For the second analysis, we developed a multivariate age-predicting model, based on GMV ROIs from HC individuals (detailed methodological procedures in section *2.4.2. Regression analysis*). This age-prediction model was then applied separately to an independent HC sample and to patients with SZ, and the study participants' Brain Age Gap Estimate (BrainAGE) scores were computed as the difference between the person’s predicted neuroanatomical and observed chronological age. For example, if a 25-year-old patient’s BrainAGE score measures five years, the neuroanatomical properties of the given brain resemble the neuroanatomical properties of a 30-year-old reference person, and hence indicate accelerated ageing in the given patient.

All GMV ROIs underwent an initial correction for total intracranial volume by dividing the value of each ROI by the total intracranial volume. The input features in both classification and regression analysis were GMV ROI values from AAL3, Hammers, and Schaefer atlases. Additionally, each analysis was repeated with the data from individual atlases separately. All analyses were conducted using MATLAB (R2022a) and R software (v4.1) within the ViPAR environment. The classification and regression ML analyses were conducted using the open-source *Machine learning in R* toolbox (mlr, v4.1) (Bischl et al., 2016).

The R scripts used for all the proof-of-concept analyses are freely available in our GitHub repository (https://github.com/adyasha95/ECNP-NNADRrepo/).

#### 2.4.1 Classification analysis

For the classification analysis, we only included sites that provided sufficient data for SZ patients (MUC: N = 156, VER: N = 118, OSL: N = 224). We matched 498 healthy individuals one-to-one for age and sex to SZ patients to avoid the problem of unbalanced samples that often occurs in ML analyses. The left-out 567 HC individuals (MUC: N = 334, VER: N = 162, OSL: N = 71) belonging to the same sites were retained to assess specificity of the classification model (Thölke et al., 2023; Chen et al., 2023).

All machine learning models were constructed within a nested repeated cross-validation (CV) framework, comprising 5 folds at both the inner (CV1, used for hyperparameter tuning) and outer CV cycles (CV2), with 5 permutations applied to each cycle. In this approach, a group of hyperparameters is chosen for every outer training set (CV2). Subsequently, the model is trained on each outer training set using the optimal hyperparameters, and its effectiveness is assessed on the outer test sets. This comprehensive approach was adopted to prevent information leakage between train and test data and enhance the generalizability of the models (Parvandeh et al., 2020).

In the ML analyses, the data preprocessing included (GBD, 2019 Mental Disorders Collaborators, 2022): feature scaling between −1 and 1 and (The Lancet Global Health, 2020) a global mean offset correction to mitigate site-effects present in the MRI ROI data (Koutsouleris et al., 2021). Concretely, we used the HC individuals to estimate site-specific means, to avoid the removal of clinically relevant effects. Then, we computed differences between the site means and the overall mean for each feature and finally subtracted these mean differences from the entire data of each respective site (HC individuals and SZ patients), in order to mean-center the data to the overall mean of the training data. All preprocessing steps were embedded within the nested cross-validation framework.

Following data preprocessing, we used the L2-Regularized L2-Loss Support Vector Classification algorithm (LiblineaRL1L2SVC) to classify HC individuals and SZ patients, and optimized the regularization parameter (*C*_*SVM*_) over the range of 2^[−6→ +4]^. Here, the decision scores from the classification models are defined as the distance between the position of the given individual in the linear kernel space and the model’s decision boundary. In this context, the magnitude of the decision scores reflects the confidence in classifying an individual towards the respective class (e.g. higher positive decision scores indicate higher confidence in the SZ class membership, while higher negative decision scores indicate high confidence in the HC class membership of a person). We assessed model performance using the true positive rate (sensitivity; SEN), true negative rate (specificity; SPE) and balanced accuracy (BAC), measured as the average between SEN and SPE. BAC was selected as the model’s optimization criterion.

In the classification analysis, we pursued three distinct approaches to effectively differentiate HC and SZ individuals. Firstly, three separate classification models were developed to classify HC and SZ individuals within each site, namely MUC, VER, and OSL. Since the model training occurred individually within each site, no site-specific corrections were performed. Individual models were further applied to the left-out HC individuals belonging to the respective sites. Secondly, a classification model was trained and cross-validated using data pooled across all sites, allowing for a comprehensive analysis of the entire dataset. This model was applied to the left-out HC individuals belonging to pooled sites. Thirdly, we employed a leave-one-site-out approach, where three classification models were trained and cross-validated using data from two sites and tested on the left-out site. Specifically, one model classified HC and SZ individuals trained on individuals from MUC and VER, which was then applied to OSL. Similarly, another model trained on individuals from MUC and OSL was applied to VER, and the third model trained on individuals from OSL and VER was applied to MUC. Furthermore, the individual models were applied to the left-out HC individuals of the left-out sites respectively to assess the model specificity. This approach enabled us to assess the generalizability of the classification models across different sites while accounting for site-specific variations.

#### 2.4.2 Regression analysis

To obtain a sample representative of all age ranges, we selected 1170 HC individuals from the total 2357 HC individuals. This selection process involved dividing the total sample into 12 age bins and sampling individuals from each bin. By doing so, we ensured that the selected sample was uniformly distributed across age groups, facilitating the construction of a normative age model that captures the variability across different age ranges. We thus avoided an underrepresentation of the tails of the age distribution which characterised the normative sample (see Figure S1 for the age distributions). We will refer to this selected HC individuals as the ‘HC normative’ sample. The remaining 1161 HC individuals were retained as an independent dataset for model validation, which we refer to as the ‘HC left-out’ sample. We used the same pre-processing steps and CV settings as previously described for the classification model in *2.5.1. Classification analysis,* and a L2-Regularized L1-Loss Support Vector Regression algorithm (LiblineaRL2L1SVR) available in the mlr R package. Next, we applied the brain-age prediction model to the HC individuals belonging to the HC left-out sample (N = 1161) as well as the patients with SZ (N = 503). We measured the precision of the model’s predictions, using the mean absolute error (MAE), Pearson correlation coefficient (*r*) and coefficient of determination (*R*^2^) between individual chronological and predicted age with MAE as the optimization criterion.

The predicted age values were corrected for their chronological age, addressing the overestimation at lower age ranges and underestimation at higher age ranges using linear regression analysis, as a common practice in BrainAGE research (Chen et al., 2022). Beta-coefficients were computed through partial correlation analysis utilizing the HC normative sample. Subsequently, these coefficients were applied to the HC left-out and SZ samples to derive corrected predicted-age values. Furthermore, the individualized BrainAGE scores were calculated by subtracting the chronological age from the corrected predicted age for both HC and SZ individuals.

Post-hoc two-sample t-tests assessed group differences between the BrainAGE scores of HC normative and left-out individuals as well as normative HC and SZ individuals at *α* = .05.

## 3 Results

### 3.1 Repository sample characteristics

Currently, the ECNP-NNADR includes 4829 participants across seven different study sites, with 51.07% of the sample consisting of HC individuals. From the entire sample, 4580 participants have MRI data available, 51.57% of which are HC individuals, 3044 participants have both clinical and imaging data and 1536 participants have only imaging data. Table 1, Table 2 provide a comprehensive overview of the demographic, clinical, and MRI data available for each site and diagnosis, respectively. The repository includes 2475 (51.25%) women, and the mean age of study participants is 41.14(SD = 16.19) years. For a visual representation of the distribution of age across sites and diagnoses, see Figure S2 and Figure S3.

Currently, the repository combines data of six clinical questionnaires, with the CTQ having the most participant responses (N = 1564), followed by the GAF (N = 1367) and PANSS (N = 1054) scales. Within the clinical data, there is a substantial overlap among the questionnaires, as depicted in Fig. 1. Specifically, there are 543 participants with both GAF and PANSS scores, while 147 participants have data for GAF, PANSS, and CTQ questionnaires. Currently, there are no participants with data for all questionnaires. For a visual representation of the distributions of clinical scale scores across sites and diagnoses, see Figure S4 and Figure S5.

### 3.2 Proof of concept analysis

For simplicity, we report the performances for models using GMV ROIs from all atlases as features in the results below, unless explicitly stated otherwise. Table 3, Table 4, Table 5 provide detailed performances of models using GMV ROIs from individual atlases and when combined from all atlases.

**Table 3:** Proof-of-concept classification analysis.

Model Performance in Classification Analysis					
Site	SEN (%)	SPE (%)	BAC (%)		SPE (%)
**All atlases**					
Munich	67.32	69.88	68.60	HC left-out	70.22
Oslo	66.86	74.45	70.65	HC left-out	65.92
Verona	49.51	52.14	50.82	HC left-out	56.66

**Schaefers**					
Munich	64.62	66.81	65.72	HC left-out	64.31
Oslo	60.29	63.57	61.93	HC left-out	43.21
Verona	50.86	51.25	51.05	HC left-out	47.94

**AAL3**					
Munich	67.46	68.46	67.96	HC left-out	66.06
Oslo	66.60	78.01	72.31	HC left-out	72.73
Verona	50.83	53.42	52.12	HC left-out	57.39

**Hammers**					
Munich	61.27	66.80	64.04	HC left-out	71.22
Oslo	65.18	77.66	71.42	HC left-out	67.49
Verona	52.57	48.99	50.78	HC left-out	54.16

*Note*. SEN = Sensitivity, SPE = Specificity, BAC = balanced accuracy.

**Table 4:** Proof-of-concept classification analysis. Out-of-sample performance of the models when applied to left-out data.

Sites	SEN (%)	SPE (%)	BAC (%)	Applied Site	SEN (%)	SPE (%)	BAC (%)
**All atlases**							

Munich + Oslo	67.63	74.63	71.13	Verona (HC matched + SZ)	21.19	78.00	49.59
				Verona (HC all + SZ)	21.53	75.34	48.43
				Verona (HC left-out)	–	73.53	–

Munich + Verona	55.68	57.46	56.57	Oslo (HC matched + SZ)	48.02	74.25	61.13
				Oslo (HC all + SZ)	48.29	73.87	61.08
				Oslo (HC left-out)	–	73.18	–

Oslo + Verona	60.13	65.80	62.97	Munich (HC matched + SZ)	45.74	75.74	60.74
				Munich (HC all + SZ)	43.08	76.73	59.91
				Munich (HC left-out)	–	76.96	–

Munich + Oslo + Verona	60.93	67.63	64.29	Munich + Oslo + Verona (HC left-out)	–	64.73	–
**Schaefers**							

Munich + Oslo	61.95	63.32	62.63	Verona (HC matched + SZ)	28.92	71.22	50.7
				Verona (HC all + SZ)	26.85	71.47	49.16
				Verona (HC left-out)	–	71.56	–

Munich + Verona	56.04	57.15	56.59	Oslo (HC matched + SZ)	47.93	66.39	57.16
				Oslo (HC all + SZ)	45.68	66.12	55.90
				Oslo (HC left-out)	–	65.52	–

Oslo + Verona	53.45	56.16	54.80	Munich (HC matched + SZ)	43.90	69.49	56.69
				Munich (HC all + SZ)	42.95	69.29	56.12
				Munich (HC left-out)	–	69.39	–

Munich + Oslo + Verona	59.11	58.39	58.75	Munich + Oslo + Verona (HC left-out)	–	55.33	–
**AAL3**							

Munich + Oslo	65.74	73.79	69.76	Verona (HC matched + SZ)	20.78	78.37	49.57
				Verona (HC all + SZ)	21.25	75.46	48.37
				Verona (HC left-out)	–	73.33	–

Munich + Verona	58.02	60.00	59.01	Oslo (HC matched + SZ)	52.27	70.88	61.57
				Oslo (HC all + SZ)	52.32	69.88	61.10
				Oslo (HC left-out)	–	67.49	–

Oslo + Verona	58.31	65.97	62.14	Munich (HC matched + SZ)	53.26	73.18	63.22
				Munich (HC all + SZ)	50.21	74.01	62.11
				Munich (HC left-out)	–	74.26	–

Munich + Oslo + Verona	60.53	68.28	64.40	Munich + Oslo + Verona (HC left-out)	–	65.53	–
**Hammers**							

Munich + Oslo	66.95	71.74	69.34	Verona (HC matched + SZ)	25.36	74.07	49.71
				Verona (HC all + SZ)	25.97	71.21	48.59
				Verona (HC left-out)	–	69.53	–

Munich + Verona	51.17	56.51	53.84	Oslo (HC matched + SZ)	57.05	67.55	62.30
				Oslo (HC all + SZ)	56.25	66.83	61.54
				Oslo (HC left-out)	–	65.24	–

Oslo + Verona	58.89	67.85	63.37	Munich (HC matched + SZ)	49.90	73.38	61.64
				Munich (HC all + SZ)	48.38	74.78	61.58
				Munich (HC left-out)	–	75.29	–

Munich + Oslo + Verona	59.73	67.26	63.50	Munich + Oslo + Verona (HC left-out)	–	66.77	–

*Note*. SEN = Sensitivity, SPE = Specificity, BAC = balanced accuracy.

**Table 5:** Proof-of-concept regression analysis.

Regression Analysis				
	R2	MAE	r	BrainAGE (mean ± SD)
**HC normative**				
All Atlases	0.77	7.16	0.88^a^	1.0907e-14 (8.90)
Schaefers	0.78	7.10	0.90^a^	6.6803e-15 (8.78)
AAL3	0.78	6.98	0.88^a^	6.0487e-15 (8.65)
Hammers	0.79	6.95	0.89^a^	7.6277e-15 (8.62)
**HC left-out**				
All Atlases	0.74	6.97	0.86^a^	0.12 (8.89)
Schaefers	0.74	7.02	0.86^a^	0.09 (8.81)
AAL3	0.78	6.35	0.89^a^	−0.11 (8.09)
Hammers	0.78	6.45	0.88^a^	−0.28 (8.27)
**SZ**				
All Atlases	0.79	7.79	0.78^a^	4.49 (8.90)
Schaefers	0.62	8.10	0.79^a^	3.85 (9.13)
AAL3	0.67	7.10	0.82^a^	3.72 (8.39)
Hammers	0.64	7.85	0.80^a^	4.11 (9.05)

*Note*. r = Correlation Coefficient measured by Pearson Correlation, MAE = Mean Absolute Error, R^2^ = Coefficient of Determination, BrainAGE = Brain age Estimation, SD = Standard Deviation, HC = Healthy Controls, SZ = Schizophrenia patients.

a P ≤ 0.001.

#### 3.2.1 Classification analysis

Four hundred ninety-eight HC individuals and four hundred ninety-eight patients diagnosed with SZ, matched based on age and sex criteria, were included in the analysis [age: mean (SD)_HC_ = 33.96 (10.30), mean (SD)_sz_ = 33.59 (10.69), *t* (df) = 0.55 (992.64), *P* > .55; sex: female (%)_HC_ = 205 (41.16%), female (%)_SZ_ = 205 (41.16), *χ2* (df) = 0, *P* = 1].

**Fig. 3:**
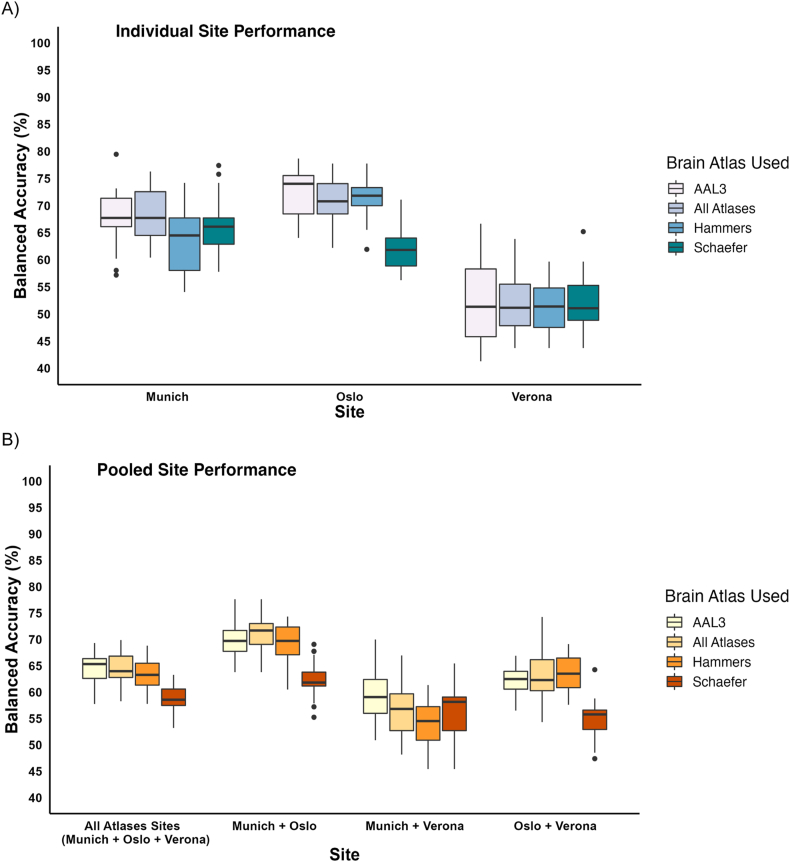
**Proof of concept analysis for the classification model.** (A) Model performance assessed using balanced accuracy for classifying SZ from HC individuals across different sites. (B) Model performance assessed using balanced accuracy for classifying SZ from HC individuals pooled from all the sites. The vertical bars represent the interquartile range of the balanced accuracies, and the dots represent outliers.

In the first approach, the model classified SZ patients and HC individuals with a BAC of 68.60% (SEN = 67.32%; SPE = 69.88%) in the MUC sample, 70.65% (SEN = 66.86%; SPE = 74.45%) in the OSL sample, and 50.82% (SEN = 49.51%; SPE = 52.14%) in the VER cohort, when using the GMV ROIs from all atlases as discriminative features (Fig. 3A). Subsequently, after applying these models to the HC left-out samples of the respective sites, we observed SPEs of 70.22%, 65.92%, and 56.66% for MUC, OSL, and VER respectively. Notably, the highest classification accuracy was achieved by the model employing ROIs extracted using the AAL3 atlas as features, particularly for individuals from the OSL site [BAC = 72.31% (SEN = 66.60%; SPE = 78.01%)].

In the second approach, the model, trained with data pooled across the three sites performed with a BAC of 64.29% (SEN = 60.93%; SPE = 67.63%). When applied to the left-out HC individuals, this model produced an SPE of 64.73%.

Moreover, in the third approach, the model trained on pooled data from OSL and VER performed with a BAC of 62.97% (SEN = 60.13%; SPE = 65.80%). In the application of this model to the matched HC and SZ individuals from the left-out site of MUC, the model yielded a BAC of 60.74% (SEN = 45.74%; SPE = 75.74%). Application of this model to the left-out HC individuals from MUC resulted in a specificity of 76.96%. The OSL + VER model demonstrated a BAC of 59.91% (SEN = 43.08%; SPE = 76.73%) across the entire MUC dataset. Furthermore, the model trained on data from MUC and VER performed with a BAC of 56.57% (SEN = 55.68%; SPE = 57.46%). When applied to the matched HC and SZ individuals from the left-out site of OSL, this model achieved a BAC of 61.13% (SEN = 48.02%; SPE = 74.25%). Application to the left-out HC individuals from OSL resulted in a specificity of 73.18%, while the entire OSL sample exhibited a BAC of 61.08% (SEN = 48.29%; SPE = 73.87%). Lastly, the model trained on the pooled data from MUC and OSL exhibited a BAC of 71.13% (SEN = 67.63%; SPE = 74.63%). When applied to the matched HC and SZ individuals from the left-out site of VER, the model yielded a BAC of 49.59% (SEN = 21.19%; SPE = 78.00%). On the left-out HC individuals from VER, the model achieved a specificity of 73.53%, and a BAC of 48.43% (SEN = 21.53%; SPE = 75.34.93%) when applied to the matched HC, SZ, and left-out HC individuals. Detailed overview of results from the classification analysis are shown in Table 4 and Fig. 3.

#### 3.2.2 Regression analysis

**Fig. 4:**
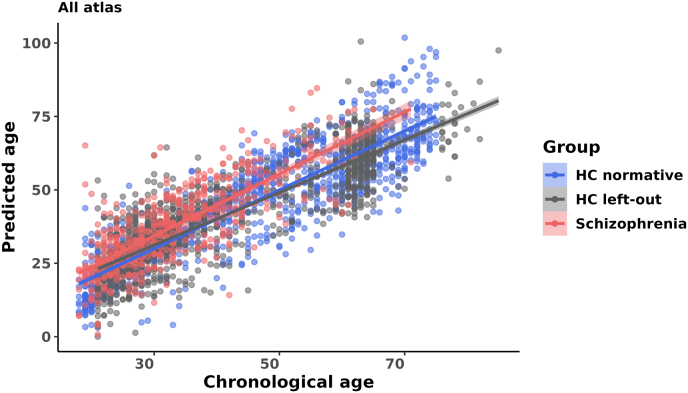
**Proof of concept analysis of the age regression model.** Chronological age v/s predicted age with a linear curve fit; the regression line with 95% confidence interval for the HC individuals in blue and the SZ group in red for the model using GMV ROI values from Schaefers, AAL3 and Hammers atlases together.

The regression model predicted age with a MAE of 7.16 years (*R*^*2*^ = 0.77, P < .001) and a mean (SD) BrainAGE of ∼0 (8.90) in the HC-normative group (Fig. 4). Upon application of this model to the left-out HC individuals, the MAE was maintained at 6.97 years (*R*^2^ = 0.74, *P* < .001) with a negligible deviation in the BrainAGE [mean (SD) = 0.12 (8.89)]. Subsequently, application of this model to patients diagnosed with SZ yielded a MAE of 7.79 (*R*^2^ = 0.79, *P* < .001) and a BrainAGE score of 4.49 (8.90). Notably, we did not observe significant BrainAGE differences between the normative and left-out HC samples [t (df) = -0.35 (2329), P = 0.73]. BrainAGE differed between HC and SZ individuals, with the SZ group exhibiting higher BrainAGE compared to both normative and the left-out HC samples (SZ vs. HC_normative_: *t* (df) = −9.46 (1671), *P* < .001; SZ vs HC_left-out_: [*t* (df) = −9.19 (1662), *P* < .001]). For detailed overview of the model performance based on individual atlas data, please refer to Table 5 and Figure S6. Additionally, for post-hoc comparisons between BrainAGE scores of HC normative, HC left-out and SZ samples, please refer to Tables S3–S5.

## 4 Discussion

Neuroimaging data has sparked significant excitement in recent years as a source of potential intermediate phenotypes of mental disorders, promising an advancement of psychiatry towards a more biologically informed framework (Chen et al., 2022; Linden, 2012).

However, the development of novel imaging-based precision psychiatry tools for addressing the worldwide burden of mental health disorders is currently hindered by the suboptimal generalizability and clinical utility of small-scale studies (Chen et al., 2023; Yarkoni, 2022). In this context, the newly established ECNP-NNADR offers a collaborative platform for addressing some of these challenges while maintaining data privacy (White et al., 2022), with an emphasis on building and validating neuroimaging-based ML models for diagnostic and prognostic applications. In the current work, we explored the opportunities the data repository offers, and addressed key issues associated with setting up a multi-site, big-data repository. To illustrate these opportunities and challenges, we conducted two proof-of-concept analyses, aimed at opening the avenue for future work leveraging the richness of the database.

First, we show that multi-atlas GMV data can separate HC individuals from SZ patients with BAC of up to 72.31% across the different cohorts in the discovery phase, and of up to BAC = 63.22% in the model validation phase, specifically when applying the model built on the Oslo and Verona data to the left-out Munich site. Our results are comparable with previous work reporting highly variable performances (ranging between ∼55 and 90%) when classifying SZ patients from HC individuals based on structural MRI data drawn from multi-site studies (Dörfel et al., 2023; Nenadić et al., 2017). Second, we built a normative age prediction model using the HC individuals available in the ECNP-NNADR. The best model performed with a MAE of 6.35 years which is comparable to previously reported age prediction models with MAE ranging between ∼2.5 and 10 years (Han et al., 2022; Dörfel et al., 2023). The application of the model to the left-out HC sample revealed BrainAGE scores that were not statistically different from those of the HC normative individuals, affirming the robustness of the model’s generalizability across heterogenous HC populations. Notably, we found accelerated brain aging in SZ patients relative to the norm, which is a consistent finding in the literature (Nenadić et al., 2017; Koutsouleris et al., 2014; Constantinides et al., 2023). In summary, our findings are consistent with prior research, underscoring the analytical potential and strengths provided by the repository.

However, the analyses also highlight obstacles hindering the translation of neuroimaging-based models into clinically reliable and generalizable tools (Dinsdale et al., 2022; Leming et al., 2023). First, the modest and variable performance of our SZ classification models across the different study cohorts emphasizes the importance of appropriately accounting for site-related heterogeneity within large-scale, multi-site datasets. Residual site effects could thus be one of the potential factors contributing to these findings, warranting further analysis. While the CAT12 processing pipeline was applied identically across sites, the optimal correction of simple and more complex scanner-related effects remains a debated research area (Bayer et al., 2022; Solanes et al., 2023). We used a simple univariate site correction method which showed good results in removing cohort effects from clinical data (Koutsouleris et al., 2021). However, scanner effects may require more elaborate procedures, such as deep learning-based methods (Bayer et al., 2022). Second, the demographic and clinical heterogeneity of subjects within and across the participating sites could have additionally diluted the neurobiological signature of SZ, leading to reduced model performance. This hypothesis is further supported by the particularly low separability of patients coming from the VER study site, who were undergoing an intensive rehabilitation program that could have potentially attenuated diagnostic brain patterns. Third, the variable accuracies of the SZ classification models when applied on the held-out validation sites revealed both over- and under-estimations of model performance in the discovery phase. Moreover, the observed variation in specificity with the increase in the validation site’s sample size, achieved by incorporating additional left-out HC samples, suggests a potential challenge to the model’s generalizability following changes in the dataset. This observation prompts consideration of the model’s susceptibility to overfitting, potentially impacting its generalizability to new samples. This further highlights the need for multiple independent validation samples for determining the true generalizability of machine learning models before clinical translation can be achieved.

Overall, the current findings illustrate equally the challenges and opportunities resulting from site-related, demographic, clinical and methodological characteristics intrinsic to ECNP-NNADR. The repository offers unique opportunities to systematically investigate novel methodological approaches which could allow overcoming these challenges, such as promising algorithms for correcting batch effects and symptom and stage-oriented clinical phenotyping (Jp et al., 2018; Hu et al., 2023). Moreover, the machine learning methodology employed here was computationally inexpensive, keeping in line with the proof-of-concept character of the current work. Exploring more elaborate deep learning, ensemble-based, and feature-selection algorithms previously reported to surpass classical methods in terms of performance and robustness constitutes a relevant future avenue to be pursued in ECNP-NNADR (Han et al., 2022; Polikar, 2012; Peng et al., 2021).

More broadly, we envision the ECNP-NNADR to facilitate scientific progress in the following ways (GBD, 2019 Mental Disorders Collaborators, 2022): development of novel differential diagnostic, prognostic, and theragnostic models with a focus on inter-site generalizability and reproducibility, by employing state-of-the-art ML methods based on thorough CV and overfitting management (The Lancet Global Health, 2020); exploration of transdiagnostic vs diagnostic-specific neurobiological pathways across major psychiatric disorders (Chodavadia et al., 2023); improvement of the clinical acceptability and feasibility of neuroimaging-based models by prioritizing model interpretability, as well as by using widely-available software and routinely employed imaging sequences; and (Fusar-Poli et al., 2016) introduction of multimodal (including both clinical and neuroimaging domains) approaches to address the disease heterogeneity commonly observed in psychiatric disorders.

Nonetheless, several limitations and avenues for further improvement of the repository are noteworthy. First, the neuroimaging data available within ECNP-NNADR is currently limited to multi-atlas grey and white matter volumetric parcellations derived using a harmonized pipeline shared across the data centers, which can be readily analyzed through the web-based ViPAR platform. In this direction, we plan to extend the repository to include voxel-level structural neuroimaging data, as well as additional neuroimaging modalities, which may provide more flexible feature extraction and modelling opportunities. Second, the repository currently only includes cross-sectional data. Recruiting more sites would allow us to extend the repository with longitudinal data sets and increase the sample sizes for the various psychiatric disorders. Third, the ECNP-NNADR is a European collaboration and, therefore, only includes individuals from this specific socio-demographic and socio-economic background. Therefore, evaluating generalizability of the results obtained in the ECNP-NNADR to other populations outside Europe necessitates future international collaborative efforts.

## 5 Conclusions

Through the ECNP-NNADR consortium, we have established a multi-site data repository consisting of clinical questionnaire and brain morphometry data belonging to patients with various psychiatric conditions, aimed at facilitating psychiatric research within the precision psychiatry framework. The two proof-of-concept analyses presented here highlight the opportunities that the ECNP-NNADR offers for state-of-the-art machine learning applications, which can propel the development of individualized diagnosis and treatment options. Ultimately, this data repository aims at facilitating international and collaborative research endeavors focused on effectively delineating the inherent heterogeneity within major psychiatric disorders.

## Acknowledgements

### Funding

NK is supported through grants from 10.13039/501100012264NIH (U01MH124639-01; ProNET), the 10.13039/100010269Wellcome Trust, the German Innovation Fund (CARE project), the German 10.13039/501100002347Federal Ministry of Education and Research (COMMITMENT and BEST projects), as well as ERA PerMed (IMPLEMENT project). AK is funded through the COMMITMENT project. CS-M and the Barcelona site were supported from Grant Nos. PI050884, PI071029, PS09/01961, PS09/01331, PI12/01306, PI13/01958, PIE14/00034, PI16/00144, PI16/00889, PI16/00950 from the Carlos III Health Institute. PB was supported by the Neuropsychological indexes and innovative treatments for major psychoses (NeuroInno). MBe was supported by PICOS - Ricerca Sanitaria Finalizzata 2004, Giunta Regionale del Veneto with a grant to Mirella Ruggeti; PREVENT & CARIVR - Fondazione Cariverona, Promoting research to improve quality of care; Sotto-obiettivo A9 “Disabilità cognitiva e comportamentale nelle demenze e nelle psicosi” and MANDRAKE - Italian ministry of Health, GR-2010-2319022 “Immune gene expression and white matter pathology in first-manic patients before and after treatment. A multimodal imaging genetic study”. GS-Imaging research initiative was supported and funded by the Welcome Trust Strategic Award “Stratifying Resilience and Depression Longitudinally” (Reference 104036/Z/14/Z). OA received support from Research Council of Norway (204966/F20, 223273, 213837); 10.13039/501100006095South-Eastern Norway Regional Health Authority (2015073, 2011-080, 2013-123); 10.13039/100007793Kristian Gerhard Jebsen Foundation. JH received support from State Research Funding, Finland, grant #P3848 (Turku TEPS 1 and 2, Turku University Hosipital). The Stradl/GenScot cohort recieved funding from 10.13039/100010269Wellcome Trust [104036/Z/14/Z, 220857/Z/20/Z, and 216767/Z/19/Z], 10.13039/100014589Chief Scientist Office of the Scottish Government Health Directorates [CZD/16/6], the Scottish Funding Council [HR03006] & 10.13039/100014013UKRI Award MR/W014386/1.

ES is supported through grants by the German 10.13039/501100002347Federal Ministry of Education and Research (COMMITMENT (grant 01ZX2204A), BEST (grant 01EK2101B), and IMPLEMENT (grant 01KU1905A) projects). We would like to thank Adriana Herrera for helping us with the data pooling and administrative work while creating the data repository.

### Role of the funding source

The ECNP Neuroimaging Network Accessible Data Repository project is a Collaborative project funded by the ECNP. The study design, data collection, analysis and publication submission process were not influenced by the funder.

### Declaration of competing interest

The authors declare the following financial interests/personal relationships which may be considered as potential competing interests: Nikolaos Koutsouleris has patent #US20160192889A1 issued to Nikolaos Koutsouleris and Eva Meisenzahl. Eva Meisenzahl has patent #US20160192889A1 issued to Nikolaos Koutsouleris and Eva Meisenzahl. As a member of Spring Health’s Scientific Advisory Board, Nikolaos Koutsouleris has advised that company on the development of tools to predict treatment outcomes for depression and psychosis. He has no equity and has received no financial compensation from this company. If there are other authors, they declare that they have no known competing financial interests or personal relationships that could have appeared to influence the work reported in this paper.
