# Supplementary material for: Towards collaborative data science in mental health research: The ECNP neuroimaging network accessible data repository
Source: Neurosci Appl. 2024 Dec 9;4:105407. doi: 10.1016/j.nsa.2024.105407 (PMC12244052; doi:10.1016/j.nsa.2024.105407)
Supplement: Multimedia component 1 [file mmc1.docx]

Supplementary material: Towards collaborative data science in mental health research: The ECNP NeuroImaging Network Accessible Data Repository

Adyasha Khuntia, MS^‡1,2,3,4^; Madalina-Octavia Buciuman, MSc^‡1,2,3,4^; John Fanning, MSc^‡1,2,3,4^; Aleks Stolicyn, PhD^5^; Clara Vetter, MSc^1,3,4,6,7^; Reetta-Liina Armio, MD^8,9^; Tiina From, PhD^8,10^; Federica Goffi, MSc^11,12^; Lisa Hahn, MSc^1,4^; Tobias Kaufmann, PhD^13,14,15^; Heikki Laurikainen, MD^8,9^; Eleonora Maggioni, PhD^11,12^; Ignacio Martinez-Zalacain, PhD^16,17^; Anne Ruef, MSc^1,4^; Mark Sen Dong, MSc^1,4^; Emanuel Schwarz, PhD^18,19,20^; Letizia Squarcina, PhD^21^; Ole Andreassen, MD^13^; Marcella Bellani, MD^22^; Paolo Brambilla, MD^21,23^; Neeltje van Haren, PhD^24,25^; Jarmo Hietala, MD^8^; Stephen M Lawrie, MD^5^; Carles Soriano-Mas, PhD^26,27,28^; Heather Whalley, PhD^5^; Maxime Taquet, PhD^29,30^; Eva Meisenzahl, MD^31^; Peter Falkai, MD^1,3,4^; Ariane Wiegand, PhD*^1,3,4^; Nikolaos Koutsouleris, MD*^1,3,4,32^ for the ECNP Neuroimaging Network^#^.

‡ These authors contributed equally to the work

* These authors contributed equally to the work

# ECNP Neuroimaging Network is described in the Acknowledgments

**Table of Contents**

[**Supplementary material: Towards collaborative data science in mental health research: The ECNP NeuroImaging Network Accessible Data Repository** 1](#_Toc158798694)

[**1.** **Supplementary Methods** 2](#_Toc158798695)

[**1.1 Clinical questionnaires available in the ECNP-NNADR 2**](#_Toc158798696)

[1.1.1 Hamilton Depression Rating Scale (HDRS) 2](#_Toc158798697)

[1.1.2 Montgomery–Åsberg Depression Rating Scale (MADRS) 2](#_Toc158798698)

[1.1.3 Childhood Trauma Questionnaire (CTQ) 3](#_Toc158798699)

[1.1.4 Global Assessment Functioning (GAF) 3](#_Toc158798700)

[1.1.5 Positive and Negative Syndrome Scale (PANSS) 3](#_Toc158798701)

[**2.** **Supplementary tables** 5](#_Toc158798702)

[**3.** **Supplementary figures** 9](#_Toc158798703)

1. Supplementary Methods
   1. Clinical questionnaires available in the ECNP-NNADR

An in-house script was developed to ensure data quality and consistency across the sites. The script verified that the clinical and MRI data were in accordance with the variable type (numeric), variable scale (continuous/categorical), and value ranges specified in the dictionaries. The script also checked that each clinical questionnaire was scored properly, meaning that the data contained the original, raw values that were matching the specified total scores. The script generated a full report that described any errors within the data so that any discrepancies could be corrected.

1.1.1 Hamilton Depression Rating Scale (HDRS)

HDRS was originally developed to assess the effectiveness of antidepressants; however, the scale gained popularity in later years as a standardized measure of depression severity (Hamilton, 1960). The original scale consists of 17 questions scored on a 5-point scale, ranging from 0 (no symptoms) to 4 (severe); these questions cover symptoms like mood, feelings of guilt, sleep quality, and anxiety (Hamilton, 1960). A 21-item version of the questionnaire also exists as an extension of the 17-item questionnaire, with the four additional questions gauging symptoms of diurnal variation, dissociation, paranoia, and obsessiveness (Hamilton, 1980). The total score of the questionnaire is a measure of overall severity of depression.

1.1.2 Montgomery–Åsberg Depression Rating Scale (MADRS)

MADRS was designed to assess depression severity while also being sensitive to treatment changes (Montgomery & Asberg, 1979). The scale consists of 10 questions scored on a 6-point scale, ranging from 0 (no symptoms) to 5 (severe). The questions measure symptoms including sadness, sleep quality, cognitive ability, and guilt. Like the HDRS, the total score of the MADRS measures the overall severity of depression.

1.1.3 Childhood Trauma Questionnaire (CTQ)

CTQ is a self-report questionnaire that assesses the presence and severity of childhood trauma (Bernstein et al., 2011). The scale assesses trauma in five different domains: emotional abuse, emotional neglect, physical abuse, physical neglect, and sexual abuse. These questions are rated on a 5-point scale, ranging from 1 (never true) to 5 (very often true). Each question belongs to one of the five domains of trauma, and the interpretation of the scoring is based on the total scores for each of the subtypes.

1.1.4 Global Assessment Functioning (GAF)

GAF is a clinician-rated scale that evaluates the overall functioning of an individual and scores individuals from 0 to 100, with higher scores indicating better functioning (Piersma & Boes, 1997). The scale uses a multi-dimensional approach as it also considers psychological, social, and occupational aspects of functioning alongside the patient’s symptoms.

1.1.5 Positive and Negative Syndrome Scale (PANSS)

PANSS assesses symptoms of schizophrenia (Kay et al., 1987). The questionnaire contains 30 questions, 16 questions related to general psychopathology symptoms like anxiety and guilt, seven questions for positive symptoms like delusions and hallucinations, and seven questions for negative symptoms like flattened affect and anhedonia. Each question is rated on a 7-point scale, ranging from 1 (absent) to 7 (extreme). The total scores of the subscales reflect symptom severity.

**1.2 Brain atlases available in the ECNP-NNADR**

**1.2.1 The automated anatomical atlas 3 (AAL3)**

The automated anatomical atlas (Tzourio-Mazoyer et al., 2002) is a parcellation of the spatially normalized single-subject high-resolution T1 volume provided by the Montreal Neurological Institute (MNI) and it’s widely used in the neuroimaging literature. Its third version (AAL3) (Rolls et al., 2020) includes a total of 166 gray matter volume cortical and subcortical regions of interest (ROI), with 26 newly added ROI in comparison to AAL2 (Rolls et al., 2015), including additional parcellations of the thalamus, nucleus accumbens, locus coeruleus, anterior cingulate cortex, substantia nigra, ventral tegmental area, red nucleus, and raphe nuclei.

1.2.2 The Schaefer-200 atlas

The Schaefer-200 atlas (Schaefer et al., 2018) is a functional atlas derived using a gradient-weighted Markov Random Field (gwMRF) model that integrates local gradient and global similarity approaches. The final functional parcellation was computed using resting-state functional MRI scans of n=1489 participants from the Genomics Superstruct Project (GSP) dataset (Holmes et al., 2015). The original set of Schaefer atlases was generated with variable numbers of ROI (100-1000) in order to allow for a flexible choice of parcellation granularity. For the ECNP-NNADR, we chose the 200 ROI parcellation as a tradeoff between a fine-grained parcellation and an appropriate data dimensionality for machine learning applications given our sample size.

1.2.3 The Hammers atlas

The Hammers brain atlas is a probabilistic, manually segmented neuroanatomical atlas derived from MRI data and expanded over time to include parcellations across gray and white matter (Hammers et al., 2003; Wild et al., 2017). The version used in this study provides 95 ROI, encompassing cortical areas, subcortical structures (e.g., hippocampus, amygdala, thalamus, basal ganglia), and detailed white matter regions. The atlas was constructed using MRI data from n=30 healthy young adult subjects, with a maximum probability map approach applied to represent the most likely anatomical region for each voxel. These updates provide a high level of spatial precision and consistency, supporting multi-site and multi-subject neuroimaging studies.

**2. Supplementary tables**

**Table S1. Overview of the 21 cohorts spanning the seven sites included in the ECNP-NNADR.** More information regarding inclusion/exclusion criteria for each cohort can be found in the reference papers provided.

| **Subcohort** | **Cohort Description** | **Funding Information** | **Reference** |
| --- | --- | --- | --- |
| **Barcelona** | | | |
| BARCENONA_PIE | Late-life MDD patients, with major depression as primary diagnosis and first episode appearing after 40 years of age. | PIE14/00034 (Carlos III Health Institute) | Neuroimage Clin  . 2020; 28:102482. doi: 10.1016/j.nicl.2020.102482. |
| BARCENONA_TEC | Cohort consisting of treatment-resistant depression patients | PS09/01961 (Carlos III Health Institute) | Transl Psychiatry  . 2017 Feb 7;7(2):e1023. doi: 10.1038/tp.2016.267 |
| BARCENONA_ANTICS | Outpatients with OCD, who satisfy DSM-IV diagnostic criteria in the absence of relevant medical, neurological, or other major psychiatric illness. | PI050884 and PI071029 (Carlos III Health Institute) | Arch Gen Psychiatry  . 2009 Nov;66(11):1189-200. doi: 10.1001/archgenpsychiatry.2009.152. |
| BARCENONA_PROV_SIMPT | OCD patients who have satisfied diagnostic criteria for at least 1 year. | PS09/01331 (Carlos III Health Institute) | Biol Psychiatry. 2013 Feb 15;73(4):321-8. doi: 10.1016/j.biopsych.2012.10.006. |
| BARCENONA_FAM_OCD | OCD patients from the Obsessive-Compulsive Disorders Unit of the Bellvitge University Hospital, who meet criteria as described in DSM-IV and confirmed by two separate interviews one month apart. | PS09/01331 (Carlos III Health Institute) | Eur Arch Psychiatry Clin Neurosci. 2014 Apr;264(3):225-33. doi: 10.1007/s00406-013-0439-7. |
| BARCENONA_HOARDING | Hoarding disorder patients, who were confirmed to have the diagnosis via the Structured Interview for Hoarding Disorder. | PI13/01958 (Carlos III Health Institute) | Psychol Med  . 2020 Mar;50(4):666-673. doi: 10.1017/S0033291719000515. |
| BARCENONA_EXT_POR | OCD patients from the OCD Unit at the Bellvitge University Hospital in Barcelona. | PI16/00144 (Carlos III Health Institute) | Behav Res Ther  . 2021 Sep; 144:103927. doi: 10.1016/j.brat.2021.103927. |
| BARCENONA_FISAX | Patients with varying levels of trait anxiety, excluding any major mental disorders or substance abuse. | PI16/00889 (Carlos III Health Institute) | Assessment. 2021 Dec 30:10731911211065166. doi: 10.1177/10731911211065166. |
| BARCENONA_COMPULSE | OCD patients having met criteria for at least a year from the OCD clinic at the Bellvitge Hospital in Barcelona. | PI16/00950 | J Affect Disord. 2020 Apr 15; 267: 23-32. DOI: 10.1016/j.jad.2020.01.161 |
| BARCENONA_RESP_CBT | OCD patients having met criteria for at least a year from the OCD clinic at the Bellvitge Hospital in Barcelona. | PI12/01306 | J Psychiatry Neurosci  . 2017 Nov;42(6):378-385. doi: 10.1503/jpn.160215. |
| **Edinburgh** | | | |
| GS-Imaging Aberdeen Subset | Participants with and without depression coming from the larger GS:SFHS study and extensively assessed to subtype MDD on the basis of its aetiology using detailed clinical, cognitive, and brain imaging assessments. | Wellcome Trust Strategic Award | Habota et al. (2020) Wellcome Open Research  <https://wellcomeopenresearch.org/articles/4-185> |
| GS-Imaging Dundee Subset |  | Wellcome Trust Strategic Award | Habota et al. (2020) Wellcome Open Research  <https://wellcomeopenresearch.org/articles/4-185> |
| ***Milan*** | | | |
| milan_15 | Patients with Bipolar disorder from Milan and Bipolar disorder type 1 from Vancouver. Diagnosis was confirmed via the SCID-I for patients from Milan or the Mini International Neurosychiatric Interview for patients from Vancouver. |  | Altamura AC, Maggioni E, Dhanoa T, Ciappolino V, Paoli RA, Cremaschi L, Prunas C, Orsenigo G, Caletti E, Cinnante CM, Triulzi FM, Dell'Osso B, Yatham L, Brambilla P. The impact of psychosis on brain anatomy in bipolar disorder: A structural MRI study. J Affect Disord. 2018 Jun;233:100-109. doi: 10.1016/j.jad.2017.11.092. Epub 2017 Nov 29. PMID: 29223329. |
| milan_30_1 |  | Neuropsychological indexes and innovative treatments for major psychoses (NeuroInno) |  |
| milan_30_2 |  | Neuropsychological indexes and innovative treatments for major psychoses (NeuroInno) |  |
| milan_30_3 |  |  |  |
| **Munich** |  |  |  |
| A1, ADHS1, Bag1, Basis1, Coghc1, Dfg, Kata1, Libi1, PS1, Sb1, Sch1, Trodat | Cohort consisting of patients with major depressive disorder, borderline personality disorder, schizophrenia, bipolar disorder, first-episode psychosis and healthy controls. |  | Koutsouleris, Nikolaos et al. “Accelerated brain aging in schizophrenia and beyond: a neuroanatomical marker of psychiatric disorders.” Schizophrenia bulletin vol. 40,5 (2014): 1140-53. doi:10.1093/schbul/sbt142 |
| **Turku** | | | |
| Turku TEPS 1 | Study consisting of patients with clinical and subclinical psychotic symptoms alongside healthy controls. Patients were recruited from either mental hospitals or psychiatric outpatient clinics. All patients were categorized as either first-episode psychosis or confirmed clinical high-risk of psychosis. |  | Salokangas, R.K.R., From, T., Ilonen, T. et al. Short-term functional outcome in psychotic patients: results of the Turku early psychosis study (TEPS). BMC Psychiatry 21, 602 (2021). https://doi.org/10.1186/s12888-021-03516-4 |
| Turku TEPS 2 |  | Grant Nos. 602152 |  |
| **Verona** | | | |
| FIRST & PICOS | FIRST:  1-year treated prevalence cohort of subjects with an ICD-10 diagnosis of schizophrenia.  PICOS:  First-episode psychosis patients from a large multi-site population-based study in the Veneto region over a 3-year period. Overall, 25 sites contributed to the PICOS study. | FIRST:  FIRST was not funded by external entities.    PICOS:  Ricerca Sanitaria Finalizzata 2004, Giunta Regionale del Veneto. | PICOS/FIRST:  Pigoni, A., Delvecchio, G., Dusi, N., Schiena, G., Andreella, A., Finos, L., Cecchetto, F., Perlini, C., Gloria Rossetti, M., Ferro, A., Bellani, M., Lasalvia, A., Ruggeri, M., Brambilla, P., & PICOS and First projects (2022). Insula volumes in first-episode and chronic psychosis: A longitudinal MRI study. Schizophrenia research, 241, 14–23. https://doi.org/10.1016/j.schres.2021.12.048 |
| PREVENT & CARIVR & MANDRAKE | PREVENT:  Longitudinal study consisting of bipolar and schizophrenia patients with the goal of uncovering neural markers involved in psychosis-onset.  CARIVR:  Cohort consisting of schizophrenia, bipolar disorder, and psychotic major depressive disorder patients.  MANDRAKE:  Bipolar patients recruited with the goal of studying the relationship between neuroimmunogenic expression and neural functioning in these patients. | PREVENT & CARIVR - Fondazione Cariverona, Promoting research to improve quality of care; Sotto-obiettivo A9 “Disabilità cognitiva e comportamentale nelle demenze e nelle psicosi”.  MANDRAKE - Italian ministry of Health, GR-2010-2319022 "Immune gene expression and white matter pathology in first-manic patients before and after treatment. A multimodal imaging genetic study”. | PREVENT/CARIVR:  Maggioni E, Crespo-Facorro B, Nenadic I, Benedetti F, Gaser C, Sauer H, Roiz-  Santiañez R, Poletti S, Marinelli V, Bellani M, Perlini C, Ruggeri M, Altamura  AC, Diwadkar VA, Brambilla P; ENPACT group. Common and distinct structural  features of schizophrenia and bipolar disorder: The European Network on  Psychosis, Affective disorders and Cognitive Trajectory (ENPACT) study. PLoS  One. 2017 Nov 14;12(11):e0188000. doi: 10.1371/journal.pone.0188000. PMID:  29136642; PMCID: PMC5685634.  Enrico P, Delvecchio G, Turtulici N, Pigoni A, Villa FM, Perlini C, Rossetti MG, Bellani M, Lasalvia A, Bonetto C, Scocco P, D'Agostino A, Torresani S, Imbesi M, Bellini F, Veronese A, Bocchio-Chiavetto L, Gennarelli M, Balestrieri M, Colombo GI, Finardi A, Ruggeri M, Furlan R, Brambilla P. Classification of Psychoses Based on Immunological Features: A Machine Learning Study in a Large Cohort of First-Episode and Chronic Patients. Schizophr Bull. 2021 Jul 8;47(4):1141-1155. doi: 10.1093/schbul/sbaa190. PMID: 33561292; PMCID: PMC8266656.    MANDRAKE:  Bellani M, Bontempi P, Zovetti N, Gloria Rossetti M, Perlini C, Dusi N,  Squarcina L, Marinelli V, Zoccatelli G, Alessandrini F, Francesca Maria Ciceri  E, Sbarbati A, Brambilla P. Resting state networks activity in euthymic bipolar  disorder. Bipolar Disord. 2020 Sep;22(6):593-601. doi: 10.1111/bdi.12900. Epub  2020 Apr 16. PMID: 32212391. |
| **Oslo** | | | |
| top15 | Cohort consisting of patients with schizophrenia, schizoaffective disorder, bipolar type I, and bipolar type II disorders. Patients recruited from 4 major hospitals in Oslo. | Funding: Research Council of Norway (204966/F20, 223273, 213837); South-Eastern Norway Regional Health Authority (2015073, 2011-080, 2013-123); Kristian Gerhard Jebsen Foundation. | Brandt CL, Eichele T, Melle I, et al. Working memory networks and activation patterns in schizophrenia and bipolar disorder: comparison with healthy controls. Br J Psychiatry. 2014;204:290–298.  Brandt CL, Kaufmann T, Agartz I, Hugdahl K, Jensen J, Ueland T, Haatveit B, Skatun KC, Doan NT, Melle I, Andreassen OA, Westlye LT. Cognitive Effort and Schizophrenia Modulate Large-Scale Functional Brain Connectivity. Schizophr Bull. 2015 Nov;41(6):1360-9. doi: 10.1093/schbul/sbv013. Epub 2015 Mar 1. PMID: 25731885; PMCID: PMC4601701.  Simonsen, Carmen et al. “Neurocognitive dysfunction in bipolar and schizophrenia spectrum disorders depends on history of psychosis rather than diagnostic group.” Schizophrenia bulletin vol. 37,1 (2011): 73-83. doi:10.1093/schbul/sbp034 |

# **Table S2. Magnetic Resonance Imagining acquisition parameters of each sub-cohort included in the ECNP-NNADR.**

| **Site** | **Subcohort** | **Scanner** | **Field strength** | **TR (ms)** | **TE (ms)** | **Flip angle** | **Voxel size (mm)** | **FOV** | **Slice num.** |
| --- | --- | --- | --- | --- | --- | --- | --- | --- | --- |
| Munich | LMU Munich cohort | Siemens Magnetom | 1.5T | 11.6 | 4.9 | NA | 0.45 x 0.45 x 1.5 | 512 x 512 | 126 |
| Barcelona | BARCENONA_PIE | Philips Ingenia | 3T | 10.46 | 4.79 | 8 | 0.75 x 0.75 x 0.75 | 320 x 320 | 233 |
| Barcelona | BARCENONA_TEC | Philips Achieva | 3T | 8.2 | 3.7 | 8 | 0.94 x 0.94 x 1.0 | 256 x 256 | 160 |
| Barcelona | BARCENONA_ANTICS | GE Signa Excite | 1.5T | 11.8 | 4.2 | 15 | 1.17 x 1.17 x 1.2 | 256 x 256 | 130 |
| Barcelona | BARCENONA_PROV_SIMPT | GE Signa Excite | 1.5T | 11.8 | 4.2 | 15 | 1.17 x 1.17 x 1.2 | 256 x 256 | 130 |
| Barcelona | BARCENONA_FAM_OCD | GE Signa Excite | 1.5T | 11.8 | 4.2 | 15 | 1.17 x 1.17 x 1.2 | 256 x 256 | 130 |
| Barcelona | BARCENONA_HOARDING | GE Signa Excite | 1.5T | 11.8 | 4.2 | 15 | 1.17 x 1.17 x 1.2 | 256 x 256 | 130 |
| Barcelona | BARCENONA_EXT_POR | Siemens Verio | 3T | 2100 | 2.67 | 9 | 1.0 x 1.0 x 1.0 | 256 x 256 | 176 |
| Barcelona | BARCENONA_FISAX | Philips Ingenia | 3T | 10.68 | 4.96 | 8 | 0.75 x 0.75 x 0.75 | 320 x 320 | 220 |
| Barcelona | BARCENONA_COMPULSE | Philips Ingenia | 3T | 10.68 | 4.96 | 8 | 0.75 x 0.75 x 0.75 | 320 x 320 | 220 |
| Barcelona | BARCENONA_RESP_CBT | Philips Ingenia | 3T | 10.68 | 4.96 | 8 | 0.75 x 0.75 x 0.75 | 320 x 320 | 220 |
| Edinburgh | GS-Imaging Aberdeen Subset | Philips Achieva | 3T | 1968 | 3.8 | 8 | 1.0 x 1.0 x 1.0 | 240 x 240 | 160 |
| Edinburgh | GS-Imaging Dundee Subset | Siemens Prisma-FIT | 3T | 1740 | 2.62 | 8 | 1.0 x 1.0 x 1.0 | 256 x 256 | 208 |
| Turku | TEPS1 | Philips Ingenia | 3T | 8.1 | 3.7 | 7 | 1.0 x 1.0 x 1.0 | 256 x 256 | 176 |
| Turku | TEPS2 | Philips Ingenia | 3T | 8.1 | 3.7 | 7 | 1.0 x 1.0 x 1.0 | 256 x 256 | 176 |
| Milan | 15 | Philips Achieva | 1.5T | 7.2 | 3.3 | 8 | 1.00 x 0.93 x 0.93 | 240 x 240 | 162 |
| Milan | 30_1 | Philips Achieva | 3T | 9.8 | 4.6 | 8 | 1.00 x 0.94 x 0.94 | NA | 185 |
| Milan | 30_2 | Philips Achieva | 3T | 7.06 | 3.4 | 8 | 1.09 x 1.04 x 1.04 | NA | 165 |
| Milan | 30_3 | Philips Achieva | 3T | NA | NA | 8 | 1.0 x 1.0 x 1.0 | NA | 160 |
| Verona | FIRST&PICOS | Siemens Allegra | 1.5T | 2060 | 3.93 | 15 | 0.46 x 0.46 x 1.25 | NA | 144 |
| Verona | PREVENT & CARIVR & MANDRAKE | Siemens Magnetom | 3T | 2300 | 3.93 | 12 | 1.0 x 1.0 x 1.0 | NA | 160 |
| Oslo | top15 | Siemens Magnetom | 1.5T | 2730 | 3.93 | 7 | 1.33 x 0.94 x 1.0 | 240 x 240 | 160 |

*Note.* TR – repetition time, TE – echo time, FOV – field of view.

**Table S3: Overview of demographic, clinical and MRI data available for each site included in the proof-of-concept classification analysis.**

|  | **Barcelona (N = 0)** | **Edinburgh (N = 0)** | **Milan (N = 0)** | **Munich**  **(N = 312)** | **Oslo**  **(N = 448)** | **Turku**  **(N = 0)** | **Verona (N = 236)** |
| --- | --- | --- | --- | --- | --- | --- | --- |
| **Age (yrs), N (mean ± SD)** | - | - | - | 312 (31.01 ± 10.02) | 448 (32.31 ±8.87) | - | 236 (40.22 ± 11.31) |
| **Sex (male, female)** | - | - | - | 232, 80 | 258, 190 | - | 96, 140 |
| **Diagnoses** | - | - | - | 156 HC, 156 SZ | 224 HC, 224 SZ |  | 118 HC, 118 SZ |
| **HDRS17, N (mean ± SD)** | - | - | - | - | - | - | - |
| **HDRS21, N (mean ± SD)** | - | - | - | - | - | - | - |
| **MADRS, N (mean ± SD)** | - | - | - | - | - | - | - |
| **CTQ, N (mean ± SD)** | - | - | - | 48 (31.85 ± 8.32) | - | - | - |
| **GAF, N (mean ± SD)** | - | - | - | 24 (37.54 ± 5.61) | 191 (47.00 ± 11.13) | - | 202 (65.56 ± 17.98) |
| **PANSS, N (mean ± SD)** | - | - | - | 152 (82.39 ± 29.70) | 168 (60.93 ± 17.92) | - | 103 (72.80 ± 29.19) |
| **MRI atlas data, N** | - | - | - | 312 | 448 | - | 236 |

*Note.* yrs = years, SD = standard deviation, SZ = schizophrenia, HC = healthy control, HDRS17 = Hamilton Depression Rating Scale (17 items), HDRS21 = Hamilton Depression Rating Scale (21 items), MADRS = Montgomery-Åsberg Depression Rating Scale, CTQ = Childhood Trauma Questionnaire, GAF = Global Assessment of Functioning, PANSS = Positive and Negative Syndrome Scale, MRI = Magnetic resonance imaging.

**Table S4: Overview of demographic, clinical and MRI variables grouped by diagnosis included in the proof-of-concept classification analysis.**

|  | **HC (N = 498)** | **SZ (N = 498)** |
| --- | --- | --- |
| **Age (yrs), N (mean ± SD)** | 498 (33.96 ± 10.30) | 498 (33.59 ± 10.69) |
| **Sex (male, female)** | 293, 205 | 293, 205 |
| **Diagnoses** | 498 HC | 498 SZ |
| **HDRS17, N (mean ± SD)** | - | - |
| **HDRS21, N (mean ± SD)** | - | - |
| **MADRS, N (mean ± SD)** | - | - |
| **CTQ, N  (mean ± SD)** | 48 (31.85 ± 8.32) | - |
| **GAF, N  (mean ± SD)** | 148 (68.99 ± 18.50) | 269 (48.00 ± 12.04) |
| **PANSS, N (mean ± SD)** | - | 423 (71.53 ± 27.13) |
| **MRI atlas data, N** | 498 | 498 |

*Note.* yrs = years, SD = standard deviation, SZ = schizophrenia, HC = healthy control, HDRS17 = Hamilton Depression Rating Scale (17 items), HDRS21 = Hamilton Depression Rating Scale (21 items), MADRS = Montgomery-Åsberg Depression Rating Scale, CTQ = Childhood Trauma Questionnaire, GAF = Global Assessment of Functioning, PANSS = Positive and Negative Syndrome Scale, MRI = Magnetic resonance imaging.

**Table S5: Overview of demographic, clinical and MRI data available for each site included in the proof-of-concept regression analysis.**

|  | **Barcelona (N = 219)** | **Edinburgh  (N = 405)** | **Milan (N = 0)** | **Munich  (N = 393)** | **Oslo  (N = 358)** | **Turku  (N = 41)** | **Verona  (N = 257)** |
| --- | --- | --- | --- | --- | --- | --- | --- |
| **Age (yrs), N (mean ± SD)** | 219 (37.19 ± 15.77) | 405 (61.44 ± 9.72) | - | 393 (33.83 ± 11.71) | 358 (34.26 ± 10.06) | 41 (27.83 ± 7.54) | 257 (40.14 ± 11.24) |
| **Sex (male, female)** | 104, 115 | 176, 229 | - | 242, 151 | 205, 153 | 24, 17 | 114, 143 |
| **Diagnoses** | 219 HC | 405 HC | - | 237 HC, 156 SZ | 134 HC, 224 SZ | 36 HC, 5 SZ | 139 HC, 118 SZ |
| **HDRS17, n (mean ± SD)** | 22 (0.64 ± 0.90) | - | - | - | - | - | 9 (8.89 ± 7.51) |
| **HDRS21, n (mean ± SD)** | - | - | - | - | - | - | 8 (14.62 ± 16.34) |
| **MADRS, n (mean ± SD)** | - | - | - | - | - | - | - |
| **CTQ, n (mean ± SD)** | - | 396 (31.57 ± 8.08) | - | 93 (31.19 ± 5.61) | - | 24 (34.29 ± 11.57) | - |
| **GAF, n (mean ± SD)** | - | - | - | 24 (37.54 ± 5.61) | 170 (47.14 ± 10.96) | 41 (85 ± 17.62) | 217 (66.90 ± 18.06) |
| **PANSS, n (mean ± SD)** | - | - | - | 152 (82.39 ± 29.70) | 168 (60.93 ± 17.92) | 26 (35.65 ± 17.42) | 103 (72.80 ± 29.19) |
| **MRI atlas data, N** | 219 | 405 | - | 393 | 358 | 41 | 257 |

*Note.* yrs = years, SD = standard deviation, SZ = schizophrenia, HC = healthy control, HDRS17 = Hamilton Depression Rating Scale (17 items), HDRS21 = Hamilton Depression Rating Scale (21 items), MADRS = Montgomery-Åsberg Depression Rating Scale, CTQ = Childhood Trauma Questionnaire, GAF = Global Assessment of Functioning, PANSS = Positive and Negative Syndrome Scale, MRI = Magnetic resonance imaging.

**Table S4: Overview of demographic, clinical and MRI variables grouped by diagnosis included in the proof-of-concept regression analysis.**

|  | **HC (N = 1170)** | **SZ (N = 503)** |
| --- | --- | --- |
| **Age (yrs), N (mean ± SD)** | 1170 (45.35 ± 16.76) | 503 (33.54 ± 10.70) |
| **Sex  (male, female)** | 1170 (569, 601) | 503 (296, 207) |
| **HDRS17, N (mean ± SD)** | 22 (0.64 ± .90) | 9 (8.89 ± 7.51) |
| **HDRS21, N (mean ± SD)** | - | 8 (14.62 ± 16.34) |
| **MADRS, N (mean ± SD)** | - | - |
| **CTQ, N  (mean ± SD)** | 510 (31.85 ± 8.32) | 3 (42.67 ± 10.69) |
| **GAF, N  (mean ± SD)** | 178 (77.44 ± 15.58) | 274 (47.93 ± 12.32) |
| **PANSS, N (mean ± SD)** | 23 (30.22 ± 0.74) | 426 (71.57 ± 27.11) |
| **MRI atlas data, N** | 1170 | 503 |

*Note.* yrs = years, SD = standard deviation, SZ = schizophrenia, HC = healthy control, HDRS17 = Hamilton Depression Rating Scale (17 items), HDRS21 = Hamilton Depression Rating Scale (21 items), MADRS = Montgomery-Åsberg Depression Rating Scale, CTQ = Childhood Trauma Questionnaire, GAF = Global Assessment of Functioning, PANSS = Positive and Negative Syndrome Scale, MRI = Magnetic resonance imaging.

**Table S5: Proof-of-concept classification analysis using logistic regression. Out-of-sample performance of the models when applied to left-out data.**

|  | **SEN (%)** | **SPE (%)** | **BAC (%)** | | **Applied Site** | **SEN (%)** | **SPE (%)** | **BAC (%)** |
| --- | --- | --- | --- | --- | --- | --- | --- | --- |
| **All atlases** | | | | | | | |  |
| Munich + Oslo | 69.05 | 73.89 | 71.47 | Verona (HC matched + SZ) | | 19.59 | 78.78 | 49.19 |
|  |  |  |  | Verona (HC all + SZ) | | 20 | 75.19 | 47.59 |
|  |  |  |  | Verona (HC left-out) | | - | 72.47 | - |
| Munich + Verona | 54.01 | 55.84 | 54.92 | Oslo (HC matched + SZ) | | 40.27 | 78.68 | 59.47 |
|  |  |  |  | Oslo (HC all +SZ) | | 40.89 | 78.36 | 59.62 |
|  |  |  |  | Oslo (HC left-out) | | - | 76.85 | - |
| Oslo + Verona | 59.94 | 61.96 | 60.95 | Munich (HC matched + SZ) | | 38.38 | 78.95 | 58.67 |
|  |  |  |  | Munich (HC all +SZ) | | 37 | 79.8 | 58.4 |
|  |  |  |  | Munich (HC left-out) | | - | 80.28 | - |
| Munich + Oslo + Verona | 63.38 | 64.54 | 63.96 | Munich + Oslo + Verona (HC left-out) | | - | 63.1 | - |
| **Schaefers** | | | | | | | |  |
| Munich + Oslo | 63.89 | 62.26 | 63.08 | Verona (HC matched + SZ) | | 72.95 | 26.54 | 49.75 |
|  |  |  |  | Verona (HC all + SZ) | | 73.16 | 25.36 | 49.26 |
|  |  |  |  | Verona (HC left-out) | | - | 72.88 | - |
| Munich + Verona | 52.04 | 51.67 | 51.86 | Oslo (HC matched + SZ) | | 73.11 | 34.71 | 53.91 |
|  |  |  |  | Oslo (HC all +SZ) | | 73.21 | 33.57 | 53.39 |
|  |  |  |  | Oslo (HC left-out) | | - | 73.01 | - |
| Oslo + Verona | 54.49 | 55.16 | 54.82 | Munich (HC matched + SZ) | | 72.56 | 36.44 | 54.5 |
|  |  |  |  | Munich (HC all +SZ) | | 73.81 | 36.46 | 55.14 |
|  |  |  |  | Munich (HC left-out) | | - | 74.47 | - |
| Munich + Oslo + Verona | 58.67 | 58.03 | 58.35 | Munich + Oslo + Verona (HC left-out) | | - | 55.41 | - |
| **AAL3** | | | | | | | |  |
| Munich + Oslo | 67.68 | 73 | 70.34 | Verona (HC matched + SZ) | | 19.08 | 78 | 48.54 |
|  |  |  |  | Verona (HC all + SZ) | | 19.93 | 75.27 | 47.6 |
|  |  |  |  | Verona (HC left-out) | | - | 73.31 | - |
| Munich + Verona | 57.08 | 60.07 | 58.58 | Oslo (HC matched + SZ) | | 73.73 | 50.02 | 61.88 |
|  |  |  |  | Oslo (HC all +SZ) | | 72.75 | 50.93 | 61.84 |
|  |  |  |  | Oslo (HC left-out) | | - | 69.75 | - |
| Oslo + Verona | 57.53 | 62.47 | 60 | Munich (HC matched + SZ) | | 73.13 | 47.62 | 60.37 |
|  |  |  |  | Munich (HC all +SZ) | | 74.81 | 45.15 | 59.98 |
|  |  |  |  | Munich (HC left-out) | | - | 75.69 | - |
| Munich + Oslo + Verona | 60.45 | 63.93 | 62.19 | Munich + Oslo + Verona (HC left-out) | | - | 63.22 | - |
| **Hammers** | | | | | | | |  |
| Munich + Oslo | 68.63 | 72.74 | 70.68 | Verona (HC matched + SZ) | | 73.93 | 25.76 | 49.85 |
|  |  |  |  | Verona (HC all + SZ) | | 70.51 | 27.08 | 48.8 |
|  |  |  |  | Verona (HC left-out) | | - | 68.07 | - |
| Munich + Verona | 54.3 | 57.23 | 55.76 | Oslo (HC matched + SZ) | | 70.02 | 56.98 | 63.5 |
|  |  |  |  | Oslo (HC all +SZ) | | 69.15 | 56.5 | 62.83 |
|  |  |  |  | Oslo (HC left-out) | | - | 65.63 | - |
| Oslo + Verona | 57.66 | 65.8 | 61.73 | Munich (HC matched + SZ) | | 72.49 | 52.56 | 62.53 |
|  |  |  |  | Munich (HC all +SZ) | | 73.4 | 51.08 | 62.24 |
|  |  |  |  | Munich (HC left-out) | | - | 74 | - |
| Munich + Oslo + Verona | 61.06 | 65.58 | 63.32 | Munich + Oslo + Verona (HC left-out) | | - | 63.8 | - |

*Note.* SEN = Sensitivity, SPE = Specificity, BAC = balanced accuracy

**Table S6: Proof-of-concept classification analysis using random forest. Out-of-sample performance of the models when applied to left-out data.**

| **Sites** | **SEN (%)** | **SPE (%)** | **BAC (%)** | **Applied Site** | **SEN (%)** | **SPE (%)** | **BAC (%)** |
| --- | --- | --- | --- | --- | --- | --- | --- |
| **All atlases** | | | | | | |  |
| Munich + Oslo | 69.05 | 73.89 | 71.47 | Verona (HC matched + SZ) | 19.59 | 78.78 | 49.19 |
|  |  |  |  | Verona (HC all + SZ) | 20 | 75.19 | 47.59 |
|  |  |  |  | Verona (HC left-out) | - | 72.47 | - |
| Munich + Verona | 54.01 | 55.84 | 54.92 | Oslo (HC matched + SZ) | 40.27 | 78.68 | 59.47 |
|  |  |  |  | Oslo (HC all +SZ) | 40.89 | 78.36 | 59.62 |
|  |  |  |  | Oslo (HC left-out) | - | 76.85 | - |
| Oslo + Verona | 59.94 | 61.96 | 60.95 | Munich (HC matched + SZ) | 38.38 | 78.95 | 58.67 |
|  |  |  |  | Munich (HC all +SZ) | 37 | 79.8 | 58.4 |
|  |  |  |  | Munich (HC left-out) | - | 80.28 | - |
| Munich + Oslo + Verona | 59.32 | 66.39 | 62.86 | Munich + Oslo + Verona (HC left-out) | - | 60.61 | - |
| **Schaefers** | | | | | | |  |
| Munich + Oslo | 54.89 | 68.47 | 61.68 | Verona (HC matched + SZ) | 67.86 | 31.29 | 49.58 |
|  |  |  |  | Verona (HC all + SZ) | 65.87 | 33.49 | 49.68 |
|  |  |  |  | Verona (HC left-out) | - | 64.4 | - |
| Munich + Verona | 53.63 | 61.6 | 57.61 | Oslo (HC matched + SZ) | 71.79 | 44.34 | 58.06 |
|  |  |  |  | Oslo (HC all +SZ) | 71.59 | 41.07 | 56.33 |
|  |  |  |  | Oslo (HC left-out) | - | 74.54 | - |
| Oslo + Verona | 53.98 | 60.48 | 57.23 | Munich (HC matched + SZ) | 65.79 | 54.79 | 60.29 |
|  |  |  |  | Munich (HC all +SZ) | 63.15 | 54.56 | 58.86 |
|  |  |  |  | Munich (HC left-out) | - | 62.24 | - |
| Munich + Oslo + Verona | 53.94 | 63.45 | 58.7 | Munich + Oslo + Verona (HC left-out) | - | 57.83 | - |
| **AAL3** | | | | | | |  |
| Munich + Oslo | 66.42 | 71.63 | 69.03 | Verona (HC matched + SZ) | 29.19 | 71.05 | 50.12 |
|  |  |  |  | Verona (HC all + SZ) | 31.19 | 68.24 | 49.71 |
|  |  |  |  | Verona (HC left-out) | - | 66.35 | - |
| Munich + Verona | 53.93 | 60.59 | 57.26 | Oslo (HC matched + SZ) | 72.25 | 55.71 | 63.98 |
|  |  |  |  | Oslo (HC all +SZ) | 70.13 | 51.91 | 61.02 |
|  |  |  |  | Oslo (HC left-out) | - | 70.08 | - |
| Oslo + Verona | 59.65 | 65.33 | 62.49 | Munich (HC matched + SZ) | 71.56 | 56.77 | 64.17 |
|  |  |  |  | Munich (HC all +SZ) | 70.19 | 54.03 | 62.11 |
|  |  |  |  | Munich (HC left-out) | - | 69.4 | - |
| Munich + Oslo + Verona | 61.37 | 66.79 | 64.08 | Munich + Oslo + Verona (HC left-out) | - | 61.64 | - |
| **Hammers** | | | | | | |  |
| Munich + Oslo | 64.32 | 70.21 | 67.26 | Verona (HC matched + SZ) | 71.42 | 31.69 | 51.56 |
|  |  |  |  | Verona (HC all + SZ) | 66.53 | 33.97 | 50.25 |
|  |  |  |  | Verona (HC left-out) | - | 64.05 | - |
| Munich + Verona | 52.18 | 60.14 | 56.16 | Oslo (HC matched + SZ) | 68.34 | 61.18 | 64.76 |
|  |  |  |  | Oslo (HC all +SZ) | 68.2 | 57.89 | 63.05 |
|  |  |  |  | Oslo (HC left-out) | - | 70.08 | - |
| Oslo + Verona | 58.37 | 66.08 | 62.22 | Munich (HC matched + SZ) | 74.21 | 55.87 | 65.04 |
|  |  |  |  | Munich (HC all +SZ) | 71.82 | 54.87 | 63.34 |
|  |  |  |  | Munich (HC left-out) | - | 70.9 | - |
| Munich + Oslo + Verona | 59.24 | 67.27 | 63.26 | Munich + Oslo + Verona (HC left-out) | - | 63.32 | - |

*Note.* SEN = Sensitivity, SPE = Specificity, BAC = balanced accuracy

**Table S7: Proof-of-concept regression analysis using random forest.**

|  | **R^2^** | **MAE** | ***r*** | **BrainAGE [mean (SD)]** |
| --- | --- | --- | --- | --- |
| **HC normative** |  |  |  |  |
| All Atlases | 0.98 | 2.02 | 0.98*** | -6.0123e-16 (2.59) |
| Schaefers | 0.97 | 2.36 | 0.98*** | 4.2754e-15 (2.92) |
| AAL3 | 0.97 | 2.29 | 0.98*** | 8.7937e-15 (2.90) |
| Hammers | 0.97 | 2.26 | 0.98*** | 2.7329e-16 (2.86) |
|  |  |  |  |  |
| **HC left-out** |  |  |  |  |
| All Atlases | 0.56 | 9.18 | 0.75*** | -4.85 (10.96) |
| Schaefers | 0.75 | 7.62 | 0.87*** | 2.13 (8.74) |
| AAL3 | 0.86 | 4.95 | 0.93*** | 1.89 (6.13) |
| Hammers | 0.79 | 6.87 | 0.89*** | 1.90 (8.00) |
|  |  |  |  |  |
| **SZ** |  |  |  |  |
| All Atlases | 0.45 | 7.13 | 0.67*** | 1.45 (9.26) |
| Schaefers | 0.60 | 8.33 | 0.77*** | 6.92 (6.88) |
| AAL3 | 0.57 | 6.26 | 0.75*** | 3.48 (7.05) |
| Hammers | 00.52.64 | 8.14 | 0.72*** | 6.18 (7.57) |

*Note. r* = Correlation Coefficient measured by Pearson Correlation, MAE = Mean Absolute Error, R^2^ = Coefficient of Determination, BrainAGE = Brain age Estimation, SD = Standard Deviation, HC = Healthy Controls, SZ = Schizophrenia patients, ***P≤0.001

**Table S8. Post-hoc comparison of brain-age deviation scores between HC-normative individuals and SZ patients.**

|  | **BrainAGE HC normative**  **[mean, (SD)]** | **BrainAGE SZ**  [**mean (SD)]** | ***T*** | ***P-*value** |
| --- | --- | --- | --- | --- |
| **All Atlases** | 1.0907e-14 (8.90) | 4.49 (8.90) | -8.82 | 9.8890e-21 |
| **Schaefers** | 6.6803e-15 (8.78) | 3.85 (9.13) | -8.12 | 8.8699e-16 |
| **AAL3** | 6.0487e-15 (8.65) | 3.72 (8.39) | -8.14 | 7.4632e-16 |
| **Hammers** | 7.6277e-15 (8.62) | 4.11 (9.05) | -8.82 | 2.9091e-18 |

*Note.* BrainAGE = Brain Age Estimation, SD = Standard Deviation, HC = Healthy Controls, SZ = Schizophrenia patients, T = t-statistics assessed by Two-sample *t*-test.

**Table S9. Post-hoc comparison of brain-age deviation scores in HC individuals**

|  | **BrainAGE**  **HC normative** [**mean (SD)]** | **BrainAGE**  **HC left-out**  [**mean (SD)]** | ***T*** | ***P-*value** |
| --- | --- | --- | --- | --- |
| **All Atlases** | 1.0907e-14 (8.90) | 0.12 (8.89) | -0.3512 | 0.73 |
| **Schaefers** | 6.6803e-15 (8.78) | 0.09 (8.81) | -0.2472 | 0.80 |
| **AAL3** | 6.0487e-15 (8.65) | -0.11 (8.09) | 0.32 | 0.75 |
| **Hammers** | 7.6277e-15 (8.62) | -0.28 (8.27) | 0.79 | 0.43 |

*Note.* BrainAGE = Brain Age Estimation, SD = Standard Deviation, HC = Healthy Control, T = t-statistics assessed by Two-sample *t*-test.

**Table S10. Post-hoc comparison of brain-age deviation scores between left-out HC individuals and patients with SZ.**

|  | **BrainAGE**  **HC left-out** [**mean (SD)]** | | **BrainAGE SZ** [**mean (SD)]** | ***T*** | ***P-*value** |
| --- | --- | --- | --- | --- | --- |
| **All Atlases** | 0.12 (8.89) | 4.49 (8.90) | | -9.19 | 1.1310e-19 |
| **Schaefers** | 0.09 (8.81) | 3.85 (9.13) | | -7.90 | 5.0313e-15 |
| **AAL3** | -0.11 (8.09) | 3.72 (8.39) | | -8.78 | 4.0264e-18 |
| **Hammers** | -0.28 (8.27) | 4.11 (9.05) | | -9.66 | 1.6296e-21 |

*Note.* BrainAGE = Brain Age Estimation, SD = Standard Deviation, HC = Healthy Controls, SZ = Schizophrenia patients, T = t-statistics assessed by Two-sample *t*-test.

# **3.** **Supplementary figures**


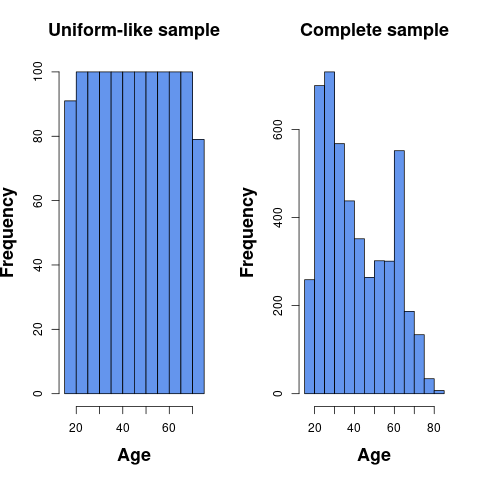


**Figure S1: Sample of HC individuals included in the regression analysis.** Histogram of the age distribution for the sample showing the full-range and uniform-like distribution of HC individuals.

**Figure S2: Distribution of age grouped by site.**

**Figure S3: Distribution of age grouped by diagnosis.**

**Figure S4: Distribution of clinical variables grouped by site.**

**Figure S5: Distribution of clinical variables grouped by diagnosis.**

**
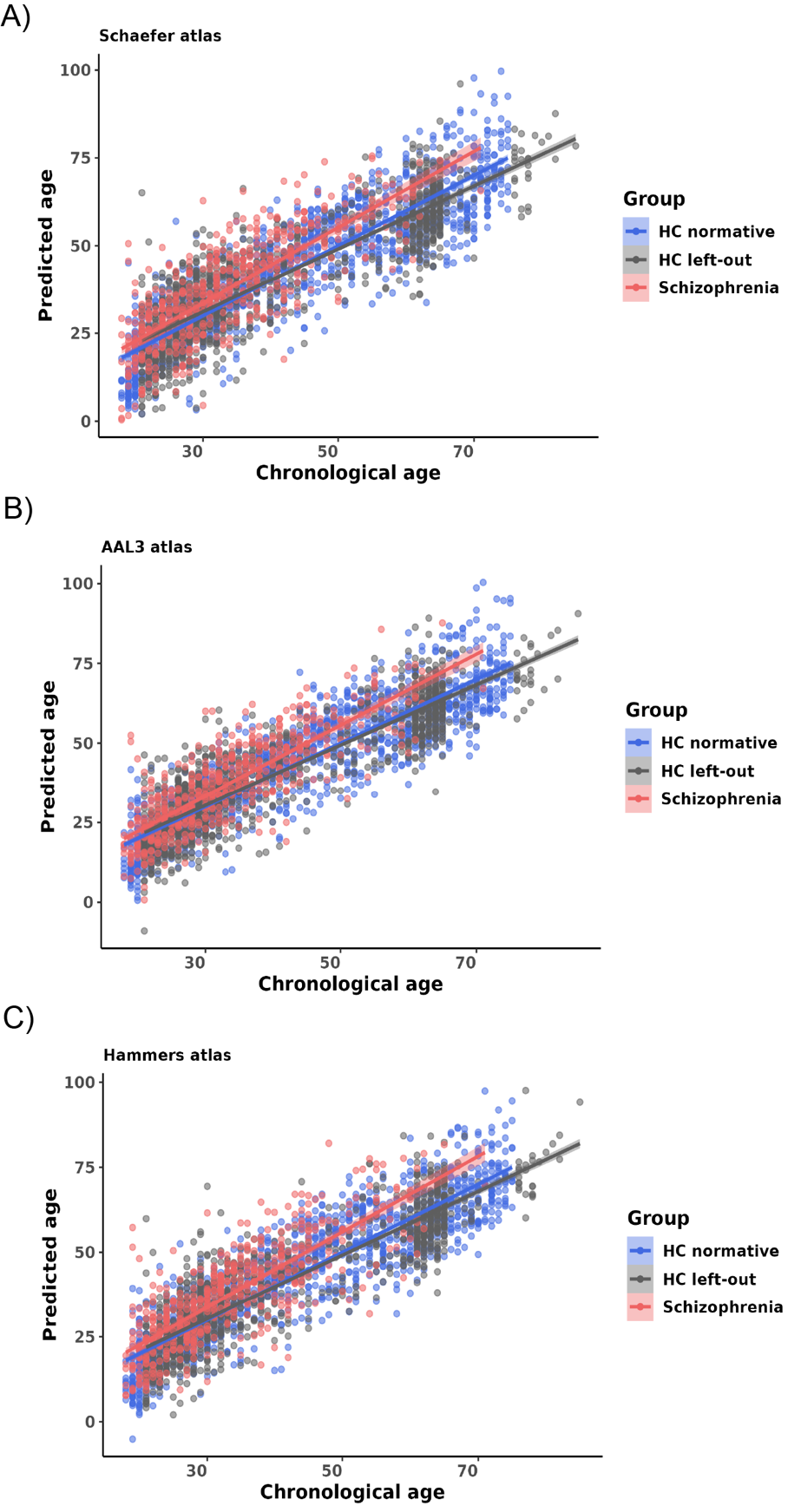

Figure S6: Results of the proof-of-concept brain age prediction analysis.** Chronological age v/s predicted age with a linear curve fit; the regression line with 95% confidence interval for the HC individuals in blue and the SZ group in red for the model using GMV ROI values from (a) Schaefers, (b) AAL3, and (c) Hammers atlases.

**References**

Bernstein, D. P., Fink, L., Handelsman, L., & Foote, J. (2011). *Childhood Trauma Questionnaire* [Dataset]. https://doi.org/10.1037/t02080-000

Hamilton, M. (1960). A RATING SCALE FOR DEPRESSION. *Journal of Neurology, Neurosurgery, and Psychiatry*, *23*(1), 56. https://doi.org/10.1136/jnnp.23.1.56

Hamilton, M. (1980). Rating depressive patients. *The Journal of Clinical Psychiatry*, *41*(12 Pt 2), 21–24.

Hammers, A., Allom, R., Koepp, M. J., Free, S. L., Myers, R., Lemieux, L., Mitchell, T. N., Brooks, D. J., & Duncan, J. S. (2003). Three-dimensional maximum probability atlas of the human brain, with particular reference to the temporal lobe. *Human Brain Mapping*, *19*(4), 224–247. https://doi.org/10.1002/hbm.10123

Holmes, A. J., Hollinshead, M. O., O’Keefe, T. M., Petrov, V. I., Fariello, G. R., Wald, L. L., Fischl, B., Rosen, B. R., Mair, R. W., Roffman, J. L., Smoller, J. W., & Buckner, R. L. (2015). Brain Genomics Superstruct Project initial data release with structural, functional, and behavioral measures. *Scientific Data*, *2*, 150031. https://doi.org/10.1038/sdata.2015.31

Kay, S. R., Fiszbein, A., & Opler, L. A. (1987). The positive and negative syndrome scale (PANSS) for schizophrenia. *Schizophrenia Bulletin*, *13*(2), 261–276. https://doi.org/10.1093/schbul/13.2.261

Montgomery, S. A., & Asberg, M. (1979). A new depression scale designed to be sensitive to change. *The British Journal of Psychiatry: The Journal of Mental Science*, *134*, 382–389. https://doi.org/10.1192/bjp.134.4.382

Piersma, H. L., & Boes, J. L. (1997). The GAF and psychiatric outcome: A descriptive report. *Community Mental Health Journal*, *33*(1), 35–41. https://doi.org/10.1023/a:1022413110345

Rolls, E. T., Huang, C.-C., Lin, C.-P., Feng, J., & Joliot, M. (2020). Automated anatomical labelling atlas 3. *NeuroImage*, *206*, 116189. https://doi.org/10.1016/j.neuroimage.2019.116189

Rolls, E. T., Joliot, M., & Tzourio-Mazoyer, N. (2015). Implementation of a new parcellation of the orbitofrontal cortex in the automated anatomical labeling atlas. *NeuroImage*, *122*, 1–5. https://doi.org/10.1016/j.neuroimage.2015.07.075

Schaefer, A., Kong, R., Gordon, E. M., Laumann, T. O., Zuo, X.-N., Holmes, A. J., Eickhoff, S. B., & Yeo, B. T. T. (2018). Local-Global Parcellation of the Human Cerebral Cortex from Intrinsic Functional Connectivity MRI. *Cerebral Cortex*, *28*(9), 3095–3114. https://doi.org/10.1093/cercor/bhx179

Tzourio-Mazoyer, N., Landeau, B., Papathanassiou, D., Crivello, F., Etard, O., Delcroix, N., Mazoyer, B., & Joliot, M. (2002). Automated anatomical labeling of activations in SPM using a macroscopic anatomical parcellation of the MNI MRI single-subject brain. *NeuroImage*, *15*(1), 273–289. https://doi.org/10.1006/nimg.2001.0978

Wild, H. M., Heckemann, R. A., Studholme, C., & Hammers, A. (2017). Gyri of the human parietal lobe: Volumes, spatial extents, automatic labelling, and probabilistic atlases. *PLOS ONE*, *12*(8), e0180866. https://doi.org/10.1371/journal.pone.0180866
